# The Gene Regulatory Network of Lens Induction Is Wired through Meis-Dependent Shadow Enhancers of *Pax6*

**DOI:** 10.1371/journal.pgen.1006441

**Published:** 2016-12-05

**Authors:** Barbora Antosova, Jana Smolikova, Lucie Klimova, Jitka Lachova, Michaela Bendova, Iryna Kozmikova, Ondrej Machon, Zbynek Kozmik

**Affiliations:** 1 Laboratory of Transcriptional Regulation, Institute of Molecular Genetics, Academy of Sciences of the Czech Republic v.v.i., Prague, Czech Republic; 2 Laboratory of Eye Biology, Institute of Molecular Genetics, Academy of Sciences of the Czech Republic v.v.i., Division BIOCEV, Prague, Czech Republic; Tel Aviv University, ISRAEL

## Abstract

Lens induction is a classical developmental model allowing investigation of cell specification, spatiotemporal control of gene expression, as well as how transcription factors are integrated into highly complex gene regulatory networks (GRNs). *Pax6* represents a key node in the gene regulatory network governing mammalian lens induction. Meis1 and Meis2 homeoproteins are considered as essential upstream regulators of *Pax6* during lens morphogenesis based on their interaction with the ectoderm enhancer (EE) located upstream of *Pax6* transcription start site. Despite this generally accepted regulatory pathway, Meis1-, Meis2- and EE-deficient mice have surprisingly mild eye phenotypes at placodal stage of lens development. Here, we show that simultaneous deletion of *Meis1* and *Meis2* in presumptive lens ectoderm results in arrested lens development in the pre-placodal stage, and neither lens placode nor lens is formed. We found that in the presumptive lens ectoderm of Meis1/Meis2 deficient embryos Pax6 expression is absent. We demonstrate using chromatin immunoprecipitation (ChIP) that in addition to EE, Meis homeoproteins bind to a remote, ultraconserved SIMO enhancer of *Pax6*. We further show, using *in vivo* gene reporter analyses, that the lens-specific activity of SIMO enhancer is dependent on the presence of three Meis binding sites, phylogenetically conserved from man to zebrafish. Genetic ablation of EE and SIMO enhancers demostrates their requirement for lens induction and uncovers an apparent redundancy at early stages of lens development. These findings identify a genetic requirement for Meis1 and Meis2 during the early steps of mammalian eye development. Moreover, they reveal an apparent robustness in the gene regulatory mechanism whereby two independent "shadow enhancers" maintain critical levels of a dosage-sensitive gene, *Pax6*, during lens induction.

## Author Summary

While significant insights into the functional role of some transcription factors during lens formation have been accomplished, much less is known about the intricate wiring of the gene regulatory network (GRN) that controls the earliest stages of lens development. Our genetic experiments presented here demonstrate a fundamental and redundant role of *Meis1* and *Meis2* genes in the process of lens induction. Furthermore, we present evidence that the robustness and dose-dependent regulation of *Pax6*, a key node of lens GRN, occurs via employment of "shadow enhancers" powered by Meis transcription factors. Combined, this study significantly extends knowledge about the genetic wiring of the earliest stages of eye development.

## Introduction

Cellular and molecular mechanisms of vertebrate lens development are objects of intense studies for many decades, reviewed in [1]. In particular, lens induction represents a classical developmental model allowing investigation of cell specification, spatiotemporal control of gene expression, as well as the integration of signaling pathways and transcription factors into highly complex gene regulatory network (GRN). At the end of neural plate formation, the vertebrate lens originates from the multipotent pre-placodal ectoderm [2, 3] through a series of cell-type specifications, governed by DNA-binding transcription factors Pax6, Six3 and Sox2, and including another transitional population of cells, the presumptive lens ectoderm (PLE). The PLE gives rise to the lens placode, readily observed as a thickening of the head surface ectoderm (SE) that is in close contact with the underlying optic vesicle, an evaginating part of the future diencephalon. Genetic dissection of lens induction has mainly focused on the function of Pax6, Six3 and Sox2, coupled with studies of BMP, retinoic acid and Wnt signaling in the surface ectoderm, neuroectoderm, and surrounding periocular mesenchyme, reviewed in [1]. *Pax6*-deficient (*Pax6* ^*Sey/Sey*^) mice are anophthalmic with eye development arrested at the optic vesicle stage [4–6]. Numerous studies have shown that Pax6 is essential for lens formation through its expression in the SE and PLE, and in the subsequent stages of lens placode formation [7–9]. In contrast, the role of Six3 and Sox2 are less clear, although it is known these factors play major roles in anterior forebrain development and optic cup formation [10–12], further enforcing *Pax6* as an ideal node to decipher genetic wiring of lens induction. Despite a well-established genetic role, much less is known about the factors operating upstream of *Pax6* and their interaction with cis-regulatory elements that direct Pax6 expression to the lens ectoderm. Since lens development is sensitive to Pax6 dosage [4] accurate regulation of Pax6 expression level during lens development is therefore of great importance.

Transcriptional control of *Pax6* gene expression is very complex and different cells and tissues choose specific promoters and distal regulatory regions from an archipelago of enhancers scattered within the large *Pax6* genomic region [13, 14]. The expression of *Pax6* in lens ectoderm was initially shown to be driven by an ectoderm enhancer (EE) located approximately 4kb upstream of the *Pax6* P0 promotor [15, 16]. However, genetic studies in which EE was inactivated provided strong evidence that EE is not the only regulatory element responsible for *Pax6* expression in the lens placode [17]. Surprisingly, detectable expression of Pax6 in lens placode of EE mutants remains. In fact, the relatively small reduction of Pax6 levels in EE mutants leads to only mild lens defects (such as a lens placode of reduced thickness and a small lens pit/vesicle) that do not phenocopy Pax6 deficiency in the PLE [7, 17] raising the possibility that additional regions compensate for the loss of EE. Genetic analysis of human aniridia patients has identified a highly conserved long-range cis-regulatory element called SIMO, located 150 kb downstream of *Pax6* [18] that can also direct transgene expression to the developing lens [19, 20] suggesting a role as a tissue-specific enhancer. Mouse-human sequence conservation around the SIMO breakpoint revealed 85% nucleotide identity over a 1400 bp fragment with 500 bp core region showing 96% identity [20]. Recently, *de novo* point mutation within the SIMO region has been identified in patient suffering aniridia. This mutation disrupts an autoregulatory *PAX6* binding site in SIMO, causing defective maintenance of PAX6 expression [19]. Remarkably, a Pax6 autoregulatory loop has also been described in the case of the EE [21]. While autoregulation of Pax6 is critical for lens cell-type identity, and represents a key mechanistic property of both *Pax6* lens enhancers, such a mechanism does not address the critical issue, namely the identification of upstream regulators of *Pax6*. To date, functional interactions of Meis1/2, Prep1, Six3, Sox2 and Oct1 have only been demonstrated at the EE [22–25].

Three amino acid loop extension (TALE) homeobox genes are evolutionarily highly conserved developmental regulators present in both vertebrate and invertebrate genomes. In vertebrates, TALE homeoproteins are represented by the Pbx and Meis/Prep subfamilies. Pbx proteins interact with Prep and Meis through a conserved amino-terminal domain while an independent protein surfaces allow Pbx to form trimeric complexes with Prep or Meis and Hox, reviewed in [26]. Prep and Meis alone preferentially bind DNA motifs with the sequence TGACAG/A, whereas Prep-Pbx and Meis-Pbx dimers bind the sequence TGATTGACAG. In mouse and human, three Meis homologs (Meis1, Meis2 and Meis3) and two homologues of Prep (Prep1 and Prep2) have been identified. Genome-wide analysis of Meis and Prep binding sites using a ChIP-seq approach have revealed their substantial specialization as well as significant regulatory coordination between these factors [27]. Biochemical and transgenic reporter studies have implicated Meis1 and Meis2 in the regulation of the EE of *Pax6* [22]. In addition, binding of Prep1 to the EE has been shown to control Pax6 levels and the timing of Pax6 activation in the developing lens [25]. However, *Meis1* knockout mice exhibit only a mild lens phenotype at later developmental stages [28]. As Meis1 and Meis2 exhibit similar expression patterns during the early stages of lens development (detailed in this study) we hypothesized that they are genetically redundant. To test this hypothesis, we have generated a *Meis2* floxed allele and subsequently investigated the effect of Meis2 and Meis1/Meis2 defficiency on lens development using a lens-specific deleter *Le-Cre* recombinase [7]. We provide genetic evidence that Meis2 alone is not essential for lens development, however combined depletion of both Meis1 and Meis2 proteins at the early stages of lens development demonstrate that Meis1/2 are redundantly required for lens placode formation. Chromatin immunoprecipitation and transgenic reporter studies further dissect the molecular mechanism of Meis-dependent regulation of *Pax6* gene expression. Deletion of SIMO region by genomic engineering *in vivo* suggests its redundancy with EE and uncovers SIMO function in lens development. Moreover, simultaneous deletion of EE and SIMO *in vivo* resulting in loss of lens formation confirms the essential role of the two *Pax6* enhancers for lens induction. Remarkably, our data demonstrate the existence of two independent and partially redundant Meis-dependent enhancers, with similar molecular architecture, involved in the regulation of Pax6 expression during lens placode formation, thereby providing an unexpected level of robustness to the system.

## Results

### Meis1 and Meis2 are expressed in overlapping pattern throughout early lens development and are redundantly required for lens induction

In this study, we sought to determine the genetic hierarchy during early lens development by investigating the role of Meis1 and Meis2 homeoproteins using knockout mice. In addition, we wanted to examine the extent of Meis-mediated regulation of the critical eye specification gene *Pax6* during lens induction. It was previously shown that specific deletion of *Pax6* in the PLE resulted in a failure of lens development from the lens placode stage onward [7]. The main prerequisite for transcriptional regulation of placodal *Pax6* expression by Meis proteins is their co-expression in the same tissue. Immunoflourescence using specific antibodies against Meis1 and Meis2 [22, 29, 30] revealed that both proteins were expressed in developing lens: in the PLE, lens placode and later in the lens pit (S1A–S1F Fig). Moreover the expression pattern of both Meis1 and Meis2 were overlapping with Pax6 expression in the PLE [31].

**Fig 1:**
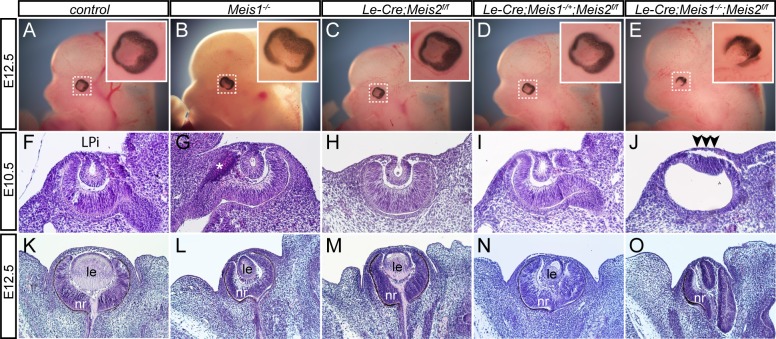
The phenotypic consequences of Meis1 and Meis2 deficiency. (**A-E**) At E12.5, external eyes of whole-mount *Meis1*^*-/-*^, *Le-Cre;Meis2*^*f/f*^, *Le-Cre;Meis1*^*+/-*^*;Meis2*^*f/f*^ mutant appear comparable to control eye, whereas the eye of *Le-Cre;Meis1*^*-/-*^*;Meis2*^*f/f*^ double mutant has abnormal shape. The insets show high magnification of eye region (boxed). (**F-O**) Hematoxylin-eosin stained parrafin sections show histology of control or mutant E10.5 and E12.5 eyes. (**F-H**, **K-M**) Formation of lens placode is followed by invagination of surface ectodem, formation of lens pit (LPi) and subsequent formation of lens in control, *Meis1*^*-/-*^ and *Le-Cre;Meis2*^*f/f*^ embryos. (**I**, **N**) One active *Meis1* allele in *Le-Cre;Meis1*^*-/+*^*; Meis2*^*f/f*^ embryos is sufficient for lens placode and lens formation. (**J**, **O**) In *Le-Cre;Meis1*^*-/-*^*;Meis2*^*f/f*^ embryos, deficient for both Meis1 and Meis2, lens development is arrested in pre-placodal stage (arrowheads). * Artefact, le-lens, nr-neural retina.

Meis1 mutants (*Meis1*^*-/-*^) do not present with arrested lens development [28]. We therefore questioned whether deletion of Meis2 may affect lens development. Accordingly, mice containing a *Meis2* floxed allele (*Meis2*^*f/f*^) were generated (S1G Fig) and [32], and subsequently zygotic *Hprt1-Cre* mice were employed to create whole-body knockout of *Meis2* (*Meis2*^*-/-*^). Meis2^-/-^ embryos displayed strong hemorrhage and other developmental defects and died by E14.5 [32]. However, lens development was not affected in these mutants (S2 Fig). To overcome the embryonic lethality of *Meis2* whole-body knockout and to conditionally inactivate Meis2 specifically in PLE from E9.0, *Le-Cre* mice [7], (S1H and S1I Fig) were crossed with *Meis2*^*f/f*^ mice. In *Le-Cre;Meis2*^*f/f*^ embryos Meis2 protein was efficiently deleted in the lens placode and surface ectoderm at E9.5 (S1J Fig). We accordingly analyzed lens development in the absence of Meis1, Meis2 or both factors. The morphology of lens development was examined at stages E10.0 and E12.5 on tissue sections stained with hematoxylin-eosin. As shown in Fig 1, both Meis1 and Meis2 deficient embryos developed beyond the lens placode stage and subsequently and invariantly formed a lens. Therefore, we decided to generate embryos simultaneously deficient for both Meis1 and Meis2 in PLE; *Le-Cre;Meis1*^*-/-*^*;Meis2*^*f/f*^ (referred thereafter as *Meis1/Meis2* double mutant). Deletion of *Meis1* and *Meis2* in the PLE of *Le-Cre;Meis1*^*-/-*^*;Meis2*^*f/f*^ embryos resulted in arrested lens development, characterized by a failure of the PLE to thicken and form the lens placode (Fig 1). Histological analysis at E12.5 confirmed an absence of lens tissue on a morphological level in all analyzed *Meis1/Meis2* double mutants, where only folded retina was present (Fig 1O). Interestingly, one functional allele of *Meis1* in *Le-Cre;Meis1*^*+/-*^*;Meis2*^*f/f*^ embryos was sufficient to ensure lens placode and later lens formation, although the lenses were typically smaller (Fig 1N). These results demonstrate a requirement for Meis proteins during lens placode and subsequent lens formation.

### Meis proteins are required for Pax6 expression in the presumptive lens ectoderm

**Fig 2:**
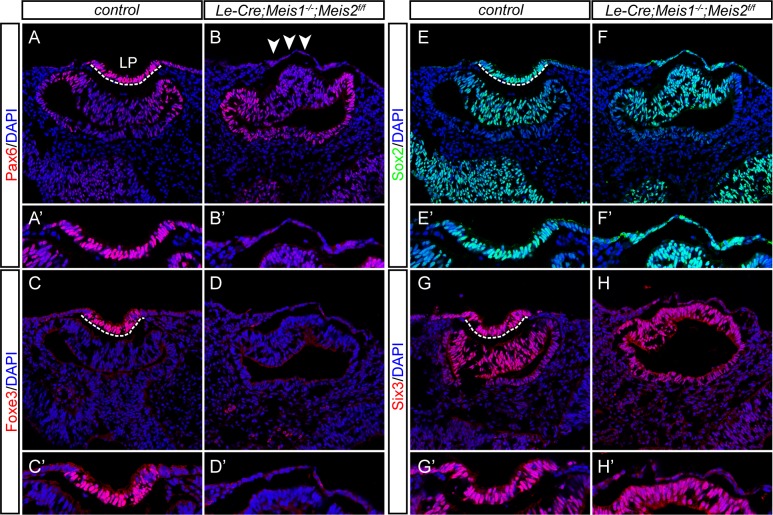
The expression of lens placode-specific transcription factors is disturbed in *Meis1/Meis2* double mutants. (**A-H‘**) Cryosections from E10.0 control and *Le-Cre;Meis1*^*-/-*^*;Meis2*^*f/f*^ embryos stained with antibody as indicated and nuclei counterstained with DAPI. (**B, B‘**) Pax6 is not detected in lens surface ectoderm of *Le-Cre;Meis1*^*-/-*^*;Meis2*^*f/f*^ embryos (arrowheads) and (**D, D‘**) expression of the lens differentiation gene *Foxe3* is not initiated. (**F, F‘**) Sox2 is detected in PLE of *Meis1/Meis2* double mutants, althouth it failed to thicken. (**H, H‘**) Finally, expression of Six3 is decreased compared to control. Lens placode (LP) is indicated by dashed line. (**A‘-H‘**) For clearer examination, lens placode or corresponding lens surface ectoderm region is magnified and shown separately.

To determine, whether the morphological arrest of lens development was accompanied by a loss of Pax6 expression and other lens placode markers, we performed immunofluorescent marker analyses at E10.0. Strikingly, we discovered a dramatic decrease in Pax6 expression in the PLE of *Meis1/Meis2* double mutants (Fig 2A–2B’). In addition, the expression of the lens differentiating gene *Foxe3*, which is known to be highly Pax6-sensitive [33], was also not initiated (Fig 2C–2D’). Conversely, Sox2 expression persisted in the PLE of E10.0 *Meis1/Meis2* double mutants (Fig 2E–2F’), which is consistent with Pax6-independent regulation of Sox2 at the lens placode stage [34]. Finally, Six3 expression that is mutually dependent on Pax6 expression in the PLE [23, 35], was also decreased in *Meis1/Meis2* double mutants (Fig 2G–2H’). Immunofluorescent analysis of E12.5 *Meis1/Meis2* double mutant embryos also confirmed the loss of α-crystallin-positive lens tissue, Prox1-positive differentiating lens fiber cells, Foxe3-positive lens epithelial cells and γ-crystallin-positive lens fiber cells (S3 Fig). Nevertheless, the presence of Pax6 and Sox2 proteins in the neural retina, and Otx2 in the retinal pigmented epithelium suggested that the specification of these tissues was not affected by the arrest of lens development (S3 Fig). Taken together, these results demonstrate that simultaneous inactivation of Meis1 and Meis2 results in early arrest of lens development and phenocopies Pax6 deficiency in the PLE [7].

### Meis proteins bind the ultraconserved SIMO element of *Pax6 in vivo*

**Fig 3:**
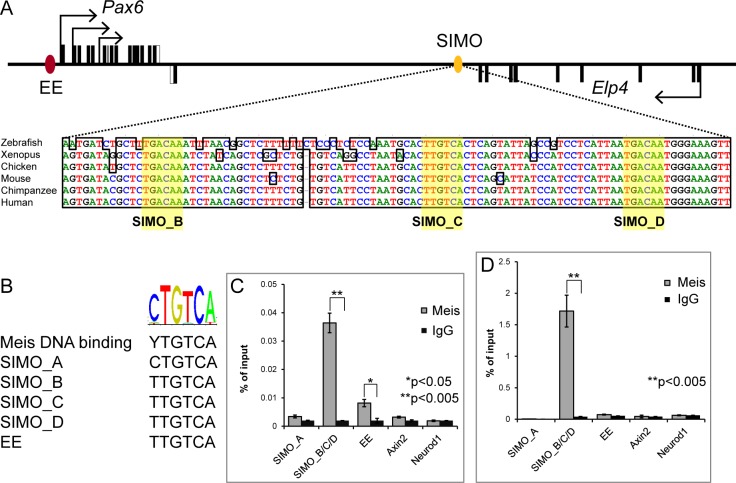
Meis proteins bind SIMO element of *Pax6 in vivo*. (**A**) Schematic representation of the *Pax6* locus, displaying the exons of *Pax6* (black boxes, top strand) and adjacent *Elp4* gene (black boxes, bottom strand). Ectoderm enhancer (EE) is indicated with red oval; SIMO enhancer is indicated with yellow oval. The detail of the part of the SIMO shows high conservation across the vertebrate species. In SIMO, five putative Meis binding sites were identified with three, SIMO_B, SIMO_C and SIMO_D (indicated with yellow color), clustered in highly conserved part of the SIMO enhancer. (**B**) The nucleotide composition of selected putative Meis binding sites found in SIMO and their comparison with Meis consensus binding site and previously identified Meis binding site in EE. (**C**, **D**) Results of chromatin immunoprecipitation of Meis-bound DNA fragments performed with the mixture of Meis1-specific and Meis2-specific antibody on chromatin prepared from E10.5 whole embryos (**C**) or αTN4 mouse lens epithelial cells (**D**) showing clear enrichment on SIMO enhancer. (**C, D**) Error bars denote SDs, *p and **p versus control using Student's *t*‐test.

A previous study has shown that Meis1 and Meis2 directly bind to the *Pax6* ectoderm enhancer (EE) and thus control Pax6 expression during early vertebrate lens induction [22]. Here we show that the simultaneous inactivation of Meis1 and Meis2 leads to the dramatic downregulation of Pax6 in PLE and arrested lens development, in a manner reminiscent of that observed in Pax6 mutants [7]. However, as deletion of the EE does not phenocopy Pax6 loss [17], we hypothesized that Meis proteins might, in addition to the EE, interact with another enhancer such as the SIMO to drive appropriate levels of Pax6 expression in the developing lens. Thus, we focused on a 1400 bp evolutionarily conserved fragment of SIMO and used chromatin immunoprecipitation (ChIP) to analyse whether Meis proteins bound the SIMO element *in vivo* (Fig 3). We initially screened the 1400 bp fragment for the presence of Meis consensus binding site sequence motif, 5’ TGACAG/A 3’ [36], (Fig 3B). In the most conserved core region of the SIMO, we identified five Meis binding sites named SIMO_A, SIMO_B, SIMO_C, SIMO_D, SIMO_E with SIMO_B/C/D clustered in DNA region of 77 bp (Fig 3A). As a positive control for Meis binding ChIP analyses, we used the EE as it has been previously described to be bound by Meis [22] and as negative controls, the Axin2 promoter and Neurod1 coding sequences were used. Chromatin immunoprecipitation was performed on wild-type E10.5 embryos and the αTN-4 cell line [37] representing a model of mouse lens epithelial cells. qRT-PCR analysis of DNA fragments immunoprecipitated with mixture of Meis1+Meis2 specific antibodies from in E10.5 embryos showed significant enrichment at the EE as well as at the SIMO_B/C/D putative Meis-interacting sites (Fig 3C). No enrichment was observed at the negative controls regions or at the predicted Meis binding site SIMO_A. Similar results were also obtained when αTN-4 cells were used for immunoprecipitation (Fig 3D). Taken together these data show that Meis proteins bind the SIMO element *in vivo* and suggest that simultaneous binding of both the EE and SIMO may be required for appropriate Pax6 expression in the early lens.

### Reporter gene analysis indicates dominant role of Meis proteins for SIMO enhancer activity

**Fig 4:**
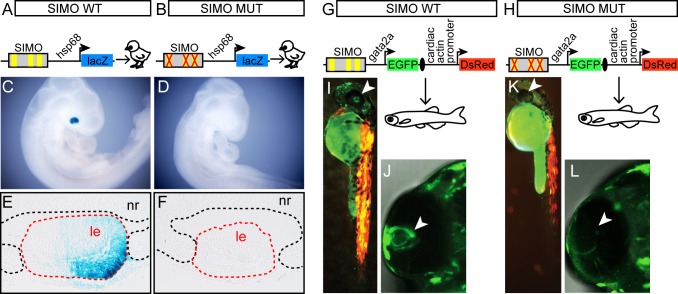
Characterization of SIMO wild-type and mutant enhancer by reporter gene assays in chick and zebrafish. (**A**, **B**) Schematic view of reporter constructs used for *in ovo* electroporation of chick embryos. Reporter constructs carry wild-type or mutant mouse SIMO element upstream of *hsp68* minimal promoter and β-galactosidase open reading frame. In mutant SIMO Meis binding sites were abolished by introduction of specific single-point mutations changing Meis recognition sequence TGACAG/A into TcACAG/A. (**C–F**) Whole-mount view or histological sections through the eye of β-galactosidase–stained chick embryos of stage HH21-22 electroporated either with (**C, E**) wild-type or with (**D, F**) mutant SIMO fragment. Positive X-gal staining correlates with the activity of reporter constructs. Wild-type SIMO fragment supports reporter construct expression in lens but not the mutant SIMO fragment. (**G, H**) Schematic view of reporter constructs used for transgenesis in zebrafish. Reporter constructs carry wild-type or mutant zebrafish SIMO element upstream of zebrafish *gata2a* minimal promoter and EGFP open reading frame. In mutant zebrafish SIMO Meis binding sites were abolished by introduction of specific single-point mutations changing Meis recognition sequence TGACAG/A into TcACAG/A. In order to control for transgenesis efficiency *in vivo* the reporter genes contain a second cassette composed of a cardiac actin promoter driving the expression of a red fluorescent protein (DsRed). EGFP and DsRed transcriptional units are separated by an insulator. (**I-L**) Wild-type SIMO enhancer activity is detected at 48 hpf (n = 160, 68% EGFP of DsRed positive), **(I, J)**, but not for the mutant construct (n = 36, 89% EGFP negative of DsRed positive) (**K, L**). LE—lens, NR—neural retina.

To test the functional significance of identified Meis interactions with the SIMO enhancer we prepared reporter gene constructs expressing lacZ gene under the control of a minimal *hsp68* promoter fused to the mouse SIMO enhancer (Fig 4A and 4B). To determine the specificity of any interactions, a single point mutation was introduced into Meis binding site that changed the recognition sequence from TGACAG/A into TcACAG/A. The same G to C mutation has previously been shown to abbrogate Meis binding and has been used in functional characterization of the EE and pancreatic enhancer in transgenic mouse models [22, 38]. In accordance with previous studies, FLAG-tagged Meis2 was able to specifically bind double-stranded oligonucleotides ancompassing wild-type Meis binding site but not its mutated version (S4 Fig). DNA constructs containing either the wild-type SIMO enhancer (SIMO WT) or the enhancer simultaneously mutated in conserved Meis binding sites SIMO_B, SIMO_C and SIMO_D (SIMO MUT), respectively, were introduced into the chick eye forming region by *in ovo* electroporation at embryonic stage HH10-11. The electroporated embryos were collected at stage HH20-21 and tested for β-galactosidase activity. As shown in Fig 4C and 4E and S5 Fig, wild-type SIMO enhancer mediated efficient expression of the lacZ reporter gene in the developing chick lens. In contrast, when all three Meis binding sites were mutated in SIMO, the lens-specific activity of the resulting reporter gene construct was abbrogated (Fig 4D and 4F and S5 Fig).

Next, we wanted to determine a possible contribution of individual Meis binding sites to SIMO enhancer activity. Mutation of SIMO_B Meis binding site alone resulted in decreased expression of reporter gene in lens as compared to wild-type SIMO, whereas simultaneous mutation of both SIMO_B and SIMO_C binding sites led to a complete loss of lens-specific expression of reporter gene (S6A Fig). These data suggest additive effect of three Meis binding sites on SIMO enhancer activity.

We noticed that Meis binding sites (sequence TGACAA in SIMO_B, SIMO_C and SIMO_D) in wild-type SIMO enhancer do not constitute the perfect match to the optimal Meis DNA-binding site motif TGACAG (http://jaspar.genereg.net/) indicating that they might represent a medium affinity sites.

In order to evaluate the functional significance of these non-optimal Meis binding sites for expression in lens we prepared reporter gene constructs expressing lacZ gene under the control of a minimal *hsp68* promoter fused to the most conserved region of mouse SIMO enhancer (hereinafter referred to as minSIMO) containing either wild-type or optimized Meis binding sites. As shown in S6B Fig, substitution of wild-type Meis binding sequence in SIMO_B, SIMO_C and SIMO_D for optimal Meis binding sequence motif resulted in higher level of reporter activity in the developing lens. These data are in accord with the key functional role of Meis proteins in SIMO regulation and indicate that strong but restricted SIMO enhancer activity relies on a cluster of three medium affinity non-optimal Meis binding sites. Notably, recent systematic study of a model enhancer shows that enhancer specificity depends on a combination of suboptimal recognition motifs having reduced binding affinities. Conversion of suboptimal binding sites to perfect matches to consensus mediates robust but ectopic patterns of gene expression [39].

Finally, in order to gain further insight into enhancer architecture we used JASPAR database (http://jaspar.genereg.net/) to screen throughout the most evolutionarily conserved core region of SIMO (minSIMO region) for consensus binding sites of additional transcription factors. We identified potential binding sites for Six3, Ets/Tead, Maf and homeodomain-containing transcription factors (S6C Fig). We performed site-directed mutagenesis of SIMO introducing dinucleotide changes in the conserved residues of the consensus binding sites (LOGOs in JASPAR database). In addition, we mutagenized an evolutionarily conserved GCTC box present in SIMO of all species analyzed in Fig 3A. Reporter gene constructs expressing lacZ gene under the control of minimal *hsp68* promoter fused either to the wild-type SIMO enhancer, or to the enhancer mutated in binding site for each particular transcription factor, were introduced into the chick eye forming region by *in ovo* electroporation at embryonic stage HH10-11. As shown in S6C Fig, none of the mutations resulted in a complete abbrogation of lens-specific reporter gene activity as did mutations in Meis binding sites SIMO_B and SIMO_C (S6A Fig). Notably, mutation of Six3 binding site resulted in decreased expression of reporter gene (S6C Fig), suggesting the requirement of similar Six3 input in SIMO enhancer as in EE [23]. Mutations in homeodomain binding sites HD1 and HD2 but not in HD3 lead to a subtle decrease of reporter activity (S6C Fig). Taken together, reporter gene assays in chick demonstrated an essential role of Meis transcription factors for SIMO enhancer activity.

Intrigued by the fact that Meis binding sites SIMO_B, SIMO_C and SIMO_D were phylogenetically conserved between mouse and zebrafish we next examined the functional significance of these sites in the context of zebrafish SIMO element. It was previously shown that the region encompassing zebrafish SIMO was able to drive expression to the lens of 48 hpf zebrafish [19]. We made a zebrafish EGFP reporter gene transgenic using wild-type and Meis-mutated versions of zebrafish SIMO element fused to minimal *gata2a* promoter (Fig 4G and 4H). In order to control for successful transgenesis and to quantitate results between the two constructs, ZED vector containing surrogate muscle-specific DsRed marker gene separated from EGFP reporter gene by an insulator was used [40]. In accordance with a previous study [19], transgenic fish carrying wild-type SIMO enhancer exhibited high level of EGFP in the lens at 48hpf (Fig 4I and 4J). In contrast, mutation of the phylogenetically conserved Meis binding sites resulted in the loss of EGFP due to the loss of lens-specific enhancer activity of SIMO while the muscle-specific surrogate reporter gene was still active (Fig 4K and 4L). These results suggest an evolutionarily conserved role of Meis proteins in the regulation of the *Pax6* SIMO enhancer. Combined, our data establish that the SIMO enhancer is a natural target of Meis1 and Meis2 and that this physical interaction conveys expression of Pax6 in developing vertebrate lens.

### Genetic ablation of SIMO and EE *in vivo*: an insight into *Pax6* enhancer redundancy

**Fig 5:**
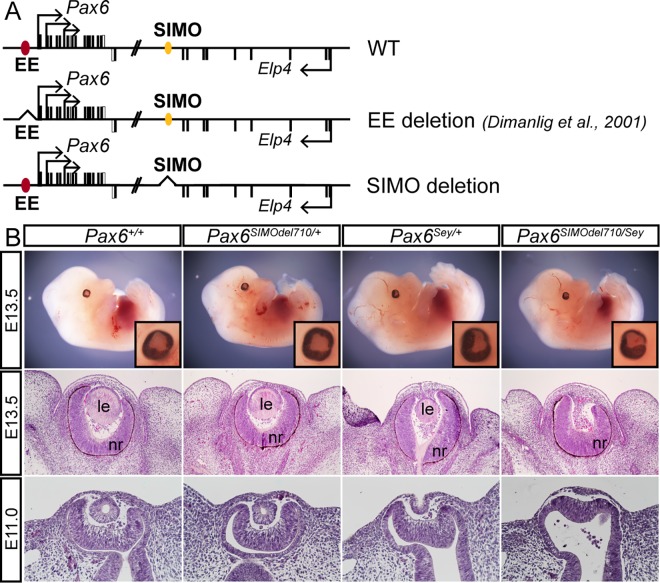
Genetic analysis of SIMO deletion *in vivo*. (**A**) Scheme of wild-type *Pax6* locus and alleles carrying EE [17] or SIMO deletion (this study). EE is indicated with red oval and SIMO with yellow oval. (**B**) Phenotypic consequences of SIMO deletion in *Pax6*^*eSIMOdel710/Sey*^ compound heterozygote mice. Whole-mount view of E13.5 embryos of the indicated genotype with eye in the inset (top panel). Histological sections through the eye demonstrating the absence of lens at E13.5 (middle panel) and arrested development prior to lens pit stage at E11.0 in *Pax6* ^*SIMOdel710/Sey*^ embryos. nr—neural retina, le-lens.

In order to get an insight into SIMO function *in vivo* we generated mice carrying deletion of its evolutionarily conserved central core. Targeted engineering of genomic DNA in *Pax6* locus was achieved using a pair of transcription activator-like effector nucleases (TALENs) designed to delete approximately 200 bp of the most evolutionarily conserved core region of SIMO (S7A Fig). Several lines of mice were established (S7B Fig) from which the line #710 designated *Pax6*^*SIMOdel710/+*^ was used for most of further studies. Enhancer region deleted in line #710 encompass Pax6 autoregulatory element and Meis1/2 binding sites SIMO_B, SIMO_C and SIMO_D, respectively, and is absolutely required for lens-specific activity based on transgenic reporter assay in chick (S7C Fig). To our surprize, mice carrying a homozygous deletion of SIMO (*Pax6*^*SIMOdel710/ SIMOdel710*^) did not manifest a major lens developmental phenotype (S7D Fig). To test whether lowering the dose of Pax6 may phenotypically uncover SIMO function during early lens development, we combined *Pax6*^*SIMOdel710/+*^ allele with *Sey* allele (Pax6 loss-of-function), (Fig 5). Under these conditions, only one allele of *Pax6* carries SIMO enhancer deletion, while the second is genetically inactive in *Sey*. Although there are several lens phenotypes associated with the complete inactivation of one *Pax6* allele in *Sey* mice, lens is always formed [5, 6], (Fig 5B). Remarkably, when the function of the second allele of *Pax6* in *Sey* mice is compromised by SIMO deletion, lens development is arrested prior to lens pit stage (Fig 5B, the bottom panel) and no lens is detected in compound *Pax6* heterozygote embryos at E13.5 (Fig 5B, the middle panel).

**Fig 6:**
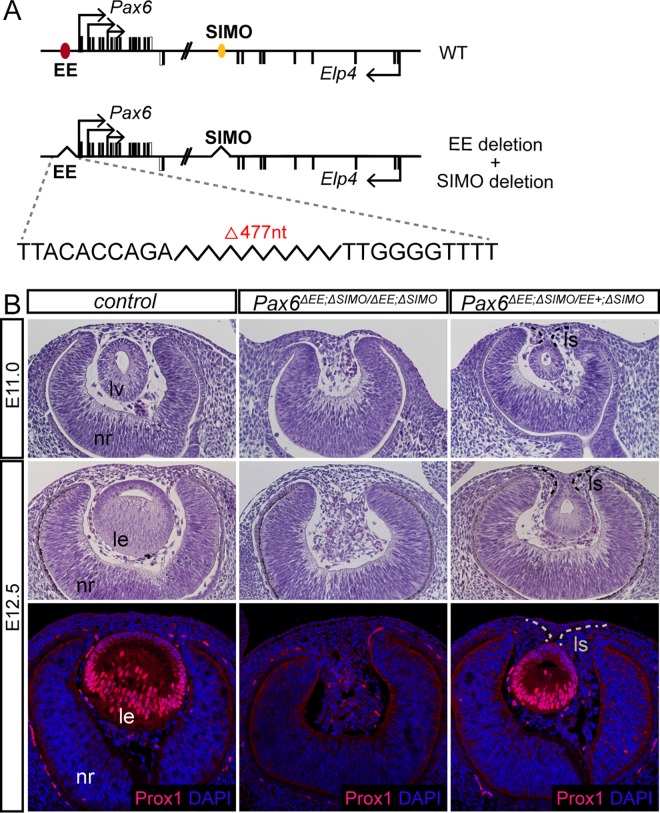
Genetic analysis of the simultaneous deletion of EE and SIMO *in vivo*. (**A**) Scheme of wild type *Pax6* locus, and allele carrying simultaneous deletion of EE and SIMO. EE is indicated with red oval and SIMO with yellow oval. The exact borders of EE deletion are specified by nucleotide sequences flanking the deletion. (**B**) Phenotypic consequences of simultaneous deletion of EE and SIMO in *Pax6*^*ΔEE;ΔSIMO/ΔEE;ΔSIMO*^ embryos. Hematoxylin and eosin stained paraffin sections demonstrating the arrested lens development prior to lens pit stage at E11.0 and absence of lens at E12.5 in *Pax6*^*ΔEE;ΔSIMO/ΔEE;ΔSIMO*^ embryos. Immunoflurescent staining for lens marker Prox1 is not detected in E12.5 *Pax6*^*ΔEE;ΔSIMO/ΔEE;ΔSIMO*^ embryos. Note that a single allele of intact EE in *Pax6*^*ΔEE;ΔSIMO/EE+; ΔSIMO*^ embryos is sufficient for lens formation albeit the resulting lens is much smaller compared to control, and lens stalk is apparent. nr—neural retina, lv – lens vesicle, le – lens, ls – lens stalk.

Finally, to demonstrate redundant role of *Pax6* enhancers EE and SIMO for lens induction, we generated mice carrying deletion of both enhancers SIMO and EE simultaneously. For that purpose, we used CRISPR/Cas9 system to delete approximately 500 bp long critical region of EE [15, 16] on the *Pax6*^*SIMOdel710/SIMOdel710*^ genetic background. Several transgenic lines of *Pax6*^*ΔEE;ΔSIMO/ ΔEE;ΔSIMO*^ mice were estabilished (hereinafter referred to as *Pax6* ^*EE/SIMO*^ double mutant), from which line containing 477bp deletion of EE simultaneously with SIMO deletion was used for further analysis (Fig 6A). Histological analysis of mice lacking all four copies of lens enhancers at E11.0 revealed arrest of lens development prior to lens pit formation (Fig 6B). Immunofluorescent staining for lens marker Prox1 at E12.5 confirmed the absence of lens tissue in *Pax6* ^*EE/SIMO*^ double mutant embryos (Fig 6B, the bottom panel). Remarkably, a single copy of a functional enhancer in *Pax6*^*ΔEE;ΔSIMO/ EE+;ΔSIMO*^ embryo was sufficient for lens induction albeit the resulting lens was much smaller at E11.0 as compared to control and lens stalk was apparent in *Pax6*^*ΔEE;ΔSIMO/ EE+;ΔSIMO*^ mice at E12.5 indicating delayed development (Fig 6B).

Genetic data indicated redundancy as well as potential additive activity of EE and SIMO. To provide further evidence that both EE and SIMO might be additively required for high level of Pax6 expression during lens induction we tested synergistic role of SIMO and EE on strength and specificity of expression of reporter genes in the developing chick lens. For that purpose we used reporter gene constructs expressing lacZ gene under the control of a minimal *hsp68* promoter fused to either SIMO alone, EE alone, or combination of both enhancers (S8 Fig). As expected, combination of full-length EE [16] with SIMO elicited stronger expression of lacZ reporter gene than did SIMO alone (S8B Fig). Similarly, combination of minimal functional EE [15] with the most conserved region of SIMO (minSIMO) ensured stronger expression than did either of the minimal enhancers alone (S8C Fig). Strong and specific reporter gene activity may also be achieved by duplication of the same type of enhancer (S8C Fig). Reporter gene assays suggest that simultaneous use of both EE and SIMO enhancers may be beneficial for achieving high-level tissue-specific *Pax6* gene expression during lens induction.

Combined, our data demonstrate simultaneous requirement of EE and SIMO *Pax6* enhancers for normal lens development and provide evidence of their apparent redundancy and synergistic activity at early stages of lens induction.

## Discussion

**Fig 7:**
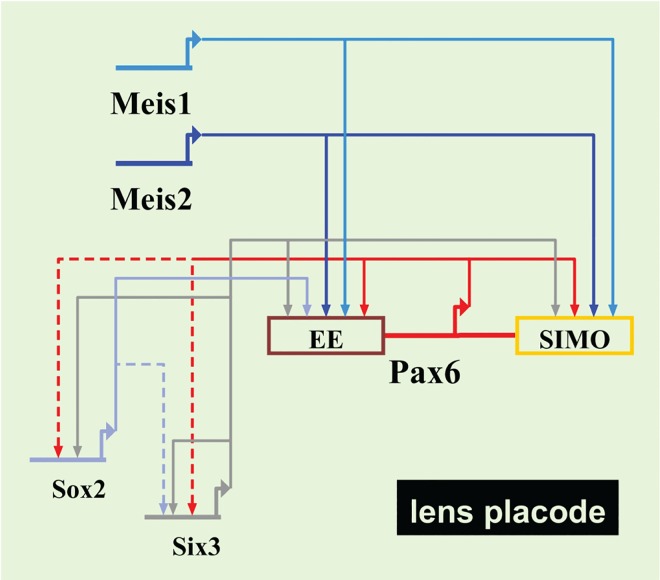
Current model of transcriptional regulatory network operating during mammalian lens induction. Direct interactions are indicated with solid lines, whereas dashed lines show possible direct interactions inferred from gain- and loss-of-function studies.

GRNs provide a system level explanation of development in terms of the genomic regulatory code [41, 42]. While significant insights into the functional role of many transcription factors during the lens placode formation have been realised, much less is known about the upstream regulation of these critical factors and the intricate wiring of the GRN that controls the earliest stages of lens development. Previous studies have shown that the GRN of mammalian lens induction is governed by a multitude of mutual cross-regulations, including the transcription factors Pax6, Six3 and Sox2 (summarized in the BioTapestry visualization Fig 7). Six3 appears to regulate the onset of Pax6 expression in the PLE while Pax6 subsequently maintains Six3 levels [23, 35, 43]. Only a small fraction of *Six3* ^*f/del*^*;Le-Cre* embryos, type III in [23], exhibit a complete arrest of lens development prior to the lens pit stage, a phenotype comparable to *Pax6* knockout phenotype, although this might be due to the inefficient deletion of *Six3*. Consequently, the level of *Six3* ablation in lens-derived tissue correlates well with the grade of phenotype and Pax6 and Sox2 downregulation [23]. Epistasis of Pax6 and Sox2 is stage-dependent. In pre-placodal ectoderm, *Pax6* and *Sox2* are regulated independently. By contrast, after the lens placode has formed, Sox2 expression is dependent on Pax6 [34]. Genetic data presented here reveal a fundamental and redundant role of Meis1 and Meis2 homeoproteins in the regulation of lens induction. Meis1 and Meis2 transcription factors have previously been identified as upstream regulators of the *Pax6* EE [22]. However, Meis1- and EE-deficient mice surprisingly do not display eye phenotypes at placodal stage of lens development [17, 28] and therefore are not comparable to that of the lens-specific ablation of *Pax6* [7]. This indicates that (i) Meis2 may compensate for the loss of Meis1, and that (ii) another *Pax6* enhancer driving expression to lens may substitute for missing EE [17, 44]. Until recently, interrogation of the combined role of Meis1/2 proteins on lens induction and Pax6 expression *in vivo* has been hampered by the lack of suitable *Meis2* knockout allele. Herein, we have conducted a comprehensive genetic analysis of Meis1 and Meis2 function in mouse to show that simultaneous depletion of Meis1 and Meis2 in the presumptive lens ectoderm results in the failure of lens placode formation and a marked reduction of Pax6 and Six3 expression in the presumptive lens areas. In contrast, expression of Sox2 is maintained in the *Meis1/Meis2* mutated ectoderm. The Meis-related TALE homeodomain protein Prep1 (also known as Pknox1) apears to control the timing of Pax6 activation and its expression level in the developing lens via direct binding to the EE [25]. The available data regarding the genetic requirement for *Prep1* suggest it has a cell-nonautonomous function in lens induction. *Prep1* trans-heterozygotes composed of a germline knockout and retroviral insertion allele (a hypomorph), respectively, demonstrate defects at the lens induction step [25]. In contrast, conditional gene targeting of *Prep1* at pre-placodal and placodal phases of lens induction using *Ap2alpha-Cre* and *Le-Cre* did not reveal any developmental phenotype [45]. We were unable to detect any changes in Prep expression using imunohistochemistry (S9 Fig), making it unlikely that the observed phenotype in *Meis1/2* double knockout mice is due to Prep1 deficiency.

Our data are consistent with the scenario in which Meis1/2 function as regulators of lens placode development primarily via activation of *Pax6* enhancers. However, it is likely that Meis1 and Meis2 regulate other factors contributing to early lens development such as the ones identified for Meis1 [46]. It was recently shown that Meis1 regulates either directly or indirectly the expression of genes involved in patterning, proliferation and differentiation of the neural retina, and that haploinsufficiency of Meis1 causes micropthalmic traits and visual impairment in adult mice [46]. Based on the fact that Marcos et al. could not detect Meis2 expression at early stages of eye development, authors considered only Meis1 function to be critical for early mouse eye development [46]. In contrast, in this study we detected Meis2 expression in early stages of lens development (S1 Fig). Furthermore, Meis2 expression is lost upon genetic ablation of *Meis2* gene (S1J Fig). This data together with the fact that only simultaneous deletion of Meis1 and Meis2 in PLE leads to an arrest of lens development in pre-placodal stage strongly suggests that both Meis1 and Meis2 are expressed and essential for early eye development. Nevertheless, it is very likely that Meis1 and Meis2 fulfill the redundant function only in specific developmental stages and processes (our data and [46]), while having many discrete functions in the embryo even within the eye development.

Mammalian eye development is highly sensitive to the levels of Pax6 as haploinsufficiency causes aniridia in humans and multiple ocular defects in mice [4, 47–50]. In contrast, increased levels of Pax6 result in various ocular abnormalities [51]. In the mammalian lens, Pax6 controls all known steps of tissue morphogenesis [7, 34, 52] but its dosage appears to be especially critical during the earliest developmental stages. The data presented here show that the molecular mechanisms of Meis1/2 regulation of *Pax6* are mediated by at least two "shadow enhancers" (Fig 7): a 3‘-located ultraconserved SIMO identified as a Meis target here, and a 5‘-located ectoderm enhancer (EE), identified as a target of TALE proteins earlier [22, 25]. The concept of the seemingly redundant "shadow enhancers" driving expression of a given gene to overlapping or identical patterns has been pioneered in *Drosophila* as a potential source of evolutionary novelty [53]. It was hypothesized that "shadow enhancers" may evolve novel binding sites and achieve new regulatory activities without disrupting the core patterning function of a developmental control gene. As cis-regulatory mutations are the main driving force of animal evolution [54, 55] buffering loss-of-function situations during enhancer evolution may be critical. "Shadow enhancers" analyzed in detail in *Drosophila* to date provide robustness and precision to the system [56–58]. A remote "shadow enhancer" identified in the human *ATOH7* gene, by virtue of its deletion in patients suffering with nonsyndromic congenital retinal nonattachment, displays identical spatiotemporal activity to the primary enhancer when tested by transgenesis [59]. Although the function of the primary and "shadow enhancer" are not firmly established, dual enhancers may reinforce *Atoh7* expression during early critical stages of eye development when retinal neurogenesis is initiated. It is tempting to speculate that the two apparently redundant distal "shadow enhancers" (EE, SIMO) ensure robust and tight regulation of *Pax6* gene expression during mammalian lens induction. In our view robustness of *Pax6* "shadow enhancer" system provides stable high level of *Pax6* gene expression and confers compensation for deleterious effects and protection to expression level fluctuations due to environmental influences. Recent systematic analysis of "shadow enhancers" during *Drosophila* mesoderm development revealed that their spatio-temporal redundancy is often partial in nature, while the non-overlapping function may explain why these enhancers are maintained within a population [60]. Reporter gene assays and genetic ablation experiments shown here provide evidence for redundant ("shadow") enhancer function of SIMO and EE selectively during early stages of lens induction. Later on the two enhancers may indeed act more independently with some overlap of transcription factor use while their distinctness is likely elicited by different sets of transcription factors co-expressed and co-bound at different times and in different combinations and stoichiometry. It is nevertheless intriguing that the two enhancers responsible for lens placode expression of Pax6 utilize similar molecular logic, namely Meis1/2-dependency ([22] and this study), Six3 regulatory input ([23] and this study) and autoregulatory function [19, 21]. Furthermore, two Meis/Prep binding sites, L1 and L2, were identified in the EE [22, 25] while at least three evolutionarily conserved Meis binding sites are present in SIMO (this study). In theory, the accumulation of homotypic binding sites may aid the enhancer robustness and may protect the enhancer from vulnerable mutations leading to the loss of responsivness to a particular transcriptional regulator. Phylogenetic footprinting and reporter gene transgenics indicate that SIMO enhancer activity in zebrafish not only depends upon Pax6 autoregulation [19] but also on functional Meis binding sites (this study). Given the profound difference in the early stages of lens development in mice (lens formed by invagination) and fish (lens arises by delamination) it is remarkable that the SIMO enhancer maintains its Meis-dependent regulation albeit not for the comparable developmental stage. In fact, SIMO enhancer becomes active in zebrafish only at 48 hours post fertilization when the lens is already formed [19]. This illustrates that species-specific adaptation of enhancer function is combined with a developmental change. It will be interesting to see if other features of SIMO regulation, such as Six3 interaction, are maintained in zebrafish. No functional data exist for the zebrafish EE, although at the sequence level this regulatory element is evolutionarily conserved from human to fish [13, 15, 25]. It remains to be seen if the evolutionary strategy of maintaining lens "shadow enhancers" in the *Pax6* locus is utilized in zebrafish, or the developmental robustness is achieved via *Pax6* gene duplication giving rise to *Pax6*.*1a* and *Pax6*.*1b* paralogues [61].

Pax6 is considered as an extreme case of an evolutionarily conserved developmental regulator promoting eye formation in vertebrates and *Drosophila* [62]. Meis genes belong to the TALE homeobox family found in genomes across all Metazoa [63]. In contrast to *Pax6*, *Homothorax*, a *Drosophila* orthologue of vertebrate *Meis/Prep* genes, suppresses eye development rather than promoting it [64]. Homothorax together with the Cut homeoprotein supresses expression of Pax6 orthologue Eyeless in the antenna disc [65]. Conversely, Sine oculis, a downstream target of Eyeless, supresses Homothorax and Cut in the eye disc thus allowing eye development to proceed [65]. The different genetic wiring of Pax6/Eyless and Meis/Homothorax in vertebrate and *Drosophila* eye developmental programs may merely reflect the vast evolutionary distance between the respective species, morphological differences in the eye types being built and a general strategy of re-purposing individual components from the common genetic toolkit during the course of evolution.

In conclusion, this study identifies a genetic requirement for Meis1 and Meis2 for early steps of mammalian eye development and reveals an apparent robustness of the gene regulatory mechanism whereby two independent "shadow enhancers" of similar molecular architecture maintain critical levels of a dosage-sensitive gene, *Pax6*, during lens induction. These results allow us to establish a genetic hierarchy during early vertebrate eye development and provide novel mechanistic insights into the regulatory logic of this process.

## Materials and Methods

### Ethics statement

Housing of mice and *in vivo* experiments were performed in compliance with the European Communities Council Directive of 24 November 1986 (86/609/EEC) and national and institutional guidelines. Animal care and experimental procedures were approved by the Animal Care Committee of the Institute of Molecular Genetics (study #174/2010). Mice were sacrificed by cervical dislocation.

### Mice

To inactivate *Meis1*, *Meis1*^*+/-*^ [28] mice were used. A conditional mutant allele of the *Meis2* gene (*Meis2*^*f/f*^) was generated by inserting loxP sites in the introns 2 and 6, flanking exons 3 and 6 in the *Meis2* gene (S1G Fig) at the Gene Targeting & Transgenic Facility, University of Connecticut, USA [32]. To generate whole-body knockout of *Meis2*, *Meis2*^*f/f*^ mice were crossed with *Hprt-Cre* mice (strain 129S1/Sv-Hprt^tm1(cre)Mnn^ /J, stock 004302, The Jackson Laboratory) that display the zygotic *Cre* recombinase activity. For specific deletion of *Meis2* in presumptive lens ectoderm, *Le-Cre* [7] mice were used. *ROSA26R* [66] and *Pax6*^*Sey-1Neu*^[4] mice (herein designated as *Pax6*^*Sey/+*^) have been described previously. SIMO enhancer was deleted using a pair of TALENs targeting sequences TCAGCCCCCACCCATACTCtcaaaaggaatgtcgTCGAGCGTCAGTGCCTGAA and TGCACTTGTCACTCAGCATTAtccatcctcattaaTGACAATGGGAAAGTTTA (recognition sequence shown in capital letters). TALENs were designed using TAL Effector Nucleotide Targeter 2.0 (https://tale-nt.cac.cornell.edu/), assembled using the Golden Gate Cloning system [67], and cloned into the ELD-KKR backbone plasmid [68]. Polyadenylated TALEN mRNAs were prepared using mMESSAGE mMACHINE T7 ULTRA Kit (Ambion) and were injected into the cytoplasm of fertilized mouse oocytes. EE [16] was deleted using CRISPR/Cas9 system. A sequence containing EE region was submitted to CRISPR Design Tool (http://crispr.mit.edu/) to select for a set of sgRNAs‘. Oligonucleotides used to make sgRNA constructs are listed in S1 Table and were cloned into pT7-gRNA (pT7-gRNA was a gift from Wenbiao Chen, Addgene plasmid # 46759). Cas9 mRNA was prepared using mMESSAGE mMACHINE T7 ULTRA Kit (Ambion) using plasmid pCS2-nCas9n (pCS2-nCas9n was a gift from Wenbiao Chen, Addgene plasmid # 47929). The sgRNAs were transcribed using MEGAshortscript kit (Ambion). A mixture of Cas9 mRNA (100ng/μl) and specific sgRNAs (25ng/μl each) was injected into the cytoplasm of fertilized mouse oocytes with homozygous or heterozygous deletion of SIMO enhancer (genetic background *Pax6*^*SIMOdel710/SIMOdel710*^ or *Pax6*^*SIMOdel710/+*^). Multiple independent lines were estabilished and the extent of EE deletion was analysed in F1 animals by DNA sequencing.

### Tissue collection, histology and immunohistochemistry

Mouse embryos were staged by designation the noon of the day when the vaginal plug was observed as embryonic day 0.5 (E0.5). Embryos of desired age were disected, fixed in 4% paraformaldehyde (PFA) from 45 minutes up to 4 hours at 4°C, washed with PBS, cryopreserved in 30% sucrose and frozen in OCT (Sakura). The cryosections (10–12 μm) were permeabilized with PBT (PBS with 0.1% Tween), blocked with 10% BSA in PBT and incubated with primary antibody (1% BSA in PBT) overnight at 4°C. Sections were washed with PBS, incubated with fluorescent secondary antibody (Life Technologies, 1:500) for one hour at room temperature, washed with PBS, counterstained with DAPI and mounted in Mowiol. The images were taken on Leica SP5 confocal microscope and were processed (contrast and brightness) with Adobe Photoshop. For hematoxylin-eosin staining, embryos were fixed in 8% PFA overnight, processed, embedded in paraffin, sectioned (8 μm), deparaffinized and stained. For β-galactosidase staining, embryos were fixed in 2% PFA, washed with rinse buffer (0.1 M phosphate buffer pH 7.3, 2 mM MgCl_2_, 20 mM Tris pH 7.3, 0.01% sodium deoxycholate, and 0.02% Nonidet P-40) and incubated in X-Gal staining solution (rinse buffer supplemented with 5 mM potasium ferricyanide, 5 mM potassium ferrocyanide, 20 mM Tris pH 7.3, and 1 mg/ml X-gal) at 37°C for 2 hours and at room temperature overnight shaking.

### Chromatin immunoprecipitation

For chromatin immunoprecipitation whole E10.5 embryos or murine lens epithelial cells αTN4 [37] were used. A chromatin immunoprecipitation assay was performed according to manufacturer’s protocol (Upstate Biotech) with slight modifications as previously described [69]. The assay was repeated twice for both embryonic and tissue culture samples. The immunoprecipitated DNA was analyzed by qRT-PCR.

### Electrophoretic mobility shift assay

In silico analysis to identify putative Meis binding sites in *SIMO* was performed using high-quality transcription factor binding profile database JASPAR [70]. Electrophoretic mobility shift assays (EMSAs) was performed using double-stranded oligonucleotides comprising binding sites SIMO_B. A single point mutation was introduced into binding site changing Meis recognition sequence TGACAG/A into TcACAG/A. ^32^P-labeled oligonucleotides were incubated with *in vitro*-synthesized FLAG-Meis2 (TNT Quick, Promega) in binding buffer (10 mM HEPES pH 7.9, 100 mM KCl, 1mM EDTA, 4% Ficoll, 0.05mg/mL poly-dIdC) at room temperature for 15 minutes. For supershift experiment, anti-FLAG M2 antibody was included in the binding reaction. Samples were analysed by 6% polyacrylamide gel electrophoresis and autoradiography.

### Electroporation *in ovo*

The wild-type mouse SIMO enhancer was amplified from genomic DNA using primers shown in S1 Table and introduced into the electroporation vector containing hsp68-lacZ reporter cassette [20]. Transcription factor binding sites within SIMO were mutagenized using QuickChange mutagenesis kit (Stratagene). Constructs carrying minimal EE and minimal SIMO enhancers were generated using synthetic double stranded oligonucleotides shown in S1 Table. All reporter gene constructs were verified by DNA sequencing. Brown Leghorn eggs were incubated until reaching HH10–11 stages and electroporation was performed as described [71]. The DNA mixture was injected outside of the right developing optic cup and electroporated using voltage of 12 V, length of pulse 20 ms, interval length 100 ms. The embryos were collected in stage HH20-HH21, fixed for 15 minutes in 2% formaldehyde and proceeded to X-gal staining.

### Zebrafish transgenesis

The wild-type zebrafish SIMO enhancer was introduced into ZED vector upstream of minimal *gata2a* promoter [40]. Meis binding sites within SIMO were mutagenized using QuickChange mutagenesis kit (Stratagene). For transgenesis, the Tol2 transposon/transposase method [72] was used with minor modifications. A mixture containing 30 ng/μl of transposase mRNA, 30 ng/μl of Qiagen column purified DNA, and 0.05% phenol red was injected in the cell of one-cell stage embryos. Embryos were raised at 28.5 ^o^C and staged by hours post fertilization (hpf). Embryos selected for imaging were anaesthetised with tricaine and mounted in low-melting agarose. Images were taken on Leica SP5 confocal microscope.

### Oligonucleotides and antibodies

All used oligonucleotides are listed in S1 Table. All used primary antibodies are listed in S2 Table.

## Supporting Information

S1 Fig Meis1 and Meis2 are co-expressed throughout early lens development. ***Le-Cre*-mediated Meis2 elimination from presumptive lens ectoderm.** (**A-F**) Cryosections from wild-type embryos of the indicated ages labeled for Meis1 and Meis2. (**A, B**) At E9.5, both Meis1 and Meis2 are expressed in lens placode (LP), and surrounding head surface ectoderm (SE) of wild-type embryo. Meis1 is also detected in optic vesicle (OV) and Meis2 in mesenchymal cells (MC). (**C**) At E10.5, Meis1 is present in lens pit (Lpi), surrounding SE, neural retina (NR) and retinal pigmented epithelium (RPE). (**D**) Meis2 expression is present in lens pit, retinal pigmented epithelium and weakly in neural retina. (**E**) At E11.5 Meis1 expression persists in SE, lens vesicle (LV), RPE and in some cells of NR. (**F**) Meis2 is detected in SE, LV, RPE and peripheral NR. (**G**) Schematic representation of targeted *Meis2* locus with marked positions of inserted loxP sites. (**H, I**) *Le-Cre* activity is demonstrated using the *ROSA26R* reporter mouse line. Whole-mounts or sections were stained with X-gal at E9.5 to show *Cre* activity in the eye primordium. (**J**) Left: Immunofluorescence signal showing Meis2 expression in surface ectoderm (SE) and lens placode (LP) in section of E9.5 control embryo. Right: Region with inactivated Meis2 is indicated with a dashed line in section of E9.5 *Meis2* mutant.
(TIF)

S2 Fig *Meis2* whole-body knockout embryos do not exhibit lens phenotype at E12.5. (**A**, **B**) External eye of E12.5 *Meis2*^*-/-*^ embryo appears comparable to control eye (magnification of eye in insets). (**C, D**) Hematoxylin-eosin-stained sections at E12.5 do not demonstrate any obvious changes of lens size or morphology in *Meis2*^*-/-*^ embryos. le – lens.
(TIF)

S3 Fig Lens specific proteins are not present in E12.5 *Meis1/Meis2* double mutant embryos. (**A-L**) Cryosections from E12.5 control and *Le-Cre;Meis1*^*-/-*^*;Meis2*^*f/f*^ embryos stained with antibody as indicated, and nuclei counterstained with DAPI. (**B**) In *Meis1/Meis2* double mutants expression of Pax6 is maintained only in neural retina and retinal pigmented epithelium (RPE), since lens is not formed.(**D, F, H, J**) Note, that lens specific proteins (α- and γ-crystallin, Prox1, Foxe3) are not detected in sections of *Le-Cre;Meis1*^*-/-*^*;Meis2*^*f/f*^ embryos. (**L**) Two separate populations of cells expressing either neural retina (Sox2) or RPE (Otx2) specific markers are detected in *Meis1/Meis2* double mutant. Scale bars indicate 100 μm. le-lens, nr-neural retina, rpe-retinal pigmented epithelium.
(TIF)

S4 Fig Meis2 binds SIMO enhancer *in vitro* using electrophoretic mobility shift assays (EMSAs). FLAG-tagged Meis2 binds wild-type SIMO_B and can be supershifted by an anti-FLAG antibody. No interaction is detected when a single point mutation is introduced into SIMO_B binding site changing Meis recognition sequence TGACAA into TcACAA.
(TIF)

S5 Fig Overview of SIMO wild-type and mutant enhancer activity in chick. (**A**) Overview of whole-mount X-gal staining of chick embryos electroporated with reporter construct containing either wild-type or mutant SIMO fragment. (**B**) Histological sections through the eye of depicted chick embryos. (**C**) Quantification of positive and negative X-gal (lacZ) staining in electroporated chick embryos.
(TIF)

S6 Fig Characterization of SIMO enhancer mutants by reporter gene assays in chick. (**A-C**) Wholemount X-gal stained chick embryos (at HH20-21) showing the expression of lacZ reporter gene under the control of minimal *hsp68* promoter fused to wild-type or mutated mouse SIMO electroporated into chick eye forming region at developmental stage HH10-11. The numbers of embryos displaying expression pattern shown are indicated in each panel. (**A**) Contribution of individual Meis binding sites to SIMO enhancer activity. Reporter gene constructs carrying wild-type SIMO (SIMO WT), SIMO mutated in a single Meis binding site (SIMO MUT-SIMO_B), or two Meis binding sites (SIMO MUT-SIMO_BC) were used for electroporation *in ovo*. Whole-mount X-gal staining demostrate the effect of mutated Meis binding sites on expression of reporter gene. Cryosections through eye region illustrate a marked decrease of lacZ expression when a single Meis binding site (SIMO_B) was mutated, and a complete loss of lens-specific expression when two Meis binding sites (SIMO_BC) were mutated. (**B**) Optimized Meis binding sites increase the activity of SIMO enhancer. Reporter gene constructs carrying either minimal wild-type SIMO (minSIMO WT), or minimal SIMO in which natural Meis binding sites TGACAA were substituted with optimized binding sequence TGACAG (minSIMO optimalMeis) were used for electroporation *in ovo*. Whole-mount X-gal staining shows that the presence of optimized Meis binding sites in SIMO moderately increases the expression of reporter gene. (**C**) The effect of selected mutations in potential transcription factor binding sites on SIMO enhancer activity. DNA constructs containing either the wild-type SIMO (SIMO WT), or the enhancer carrying mutations in binding sites for the indicated transcription factor were used for electroporation *in ovo*. Schematic pictures of transcription factor binding motifs are taken from JASPAR database. Mutated nucleotides in binding site of each transcription factor are highlighted in small red letters. nr – neural retina, le – lens.
(TIF)

S7 Fig Generation and characterization of mice carrying SIMO deletion. (**A**) Schematic representation of the *Pax6* locus, displaying the exons of *Pax6* (black boxes, top strand) and adjacent *Elp4* gene (black boxes, bottom strand). Ectodermal enhancer (EE) is indicated with red oval; SIMO enhancer is indicated with yellow oval. The relative position of TALEN recognition sequences is shown with regards to Pax6 autoregulatory element [19], shaded grey and Meis1/2 binding sites SIMO_B, SIMO_C and SIMO_D (all shaded yellow). (**B**) Schematic representation and PCR genotyping of deletions in individual lines of mice characterized (line number is indicated in red box). (**C**) Whole-mount view of β-galactosidase–stained chick embryos of stage HH21-22 electroporated either with wild-type or with mutant SIMO carrying a deletion found in line #710. Positive X-gal staining correlates with the activity of reporter constructs. (**D**) Histological sections of E11.5 and E13.5 control and *Pax6*^*SIMOdel710/SIMOdel710*^ embryonic eyes.
(TIF)

S8 Fig Additive lens specific enhancer activity is observed for EE and SIMO. (**A**) Schematic representation of the *Pax6* locus, displaying the exons of *Pax6* (black boxes, top strand) and adjacent *Elp4* gene (black boxes, bottom strand). Ectodermal enhancer (EE) is indicated with red oval; SIMO enhancer is indicated with yellow oval. (**B**,**C**) Reporter gene constructs (depicted with schematic view) carrying either SIMO alone, EE alone, or enhancer combinations were used for electroporation to reveal impact of these *Pax6* enhancers for strength and specificity of expression. Combinations of EE and SIMO (EE + SIMO, minEE + minSIMO) ensure stronger expression of reporter gene as compared to SIMO alone or EE alone. While minimal EE (minEE) drives stronger expression of reporter gene than minimal SIMO (minSIMO), the two copies of minSIMO enhancer (minSIMO 2x) provides the strongest reporter gene expression of enhancer variants tested in this experiment. The numbers of embryos displaying expression pattern shown are indicated.
(TIF)

S9 Fig Prep expression is not changed in *Meis1/Meis2* double mutant embryos. (**A, B**) Cryosections through eye region of E10.5 control and *Le-Cre;Meis1*^*-/-*^*;Meis2*^*f/f*^ embryos stained with anti-Prep antibody, and nuclei counterstained with DAPI. *Meis1/Meis2* double mutants did not show changes in Prep expression. nr-neural retina, lpi – lens pit.
(TIF)

S1 Table Oligonucleotides. (DOCX)

S2 Table Primary antibodies. (DOCX)
