# Supplementary figures and images for: The Gene Regulatory Network of Lens Induction Is Wired through Meis-Dependent Shadow Enhancers of Pax6
Source: PLoS Genet. 2016 Dec 5;12(12):e1006441. doi: 10.1371/journal.pgen.1006441 (PMC5137874; doi:10.1371/journal.pgen.1006441)

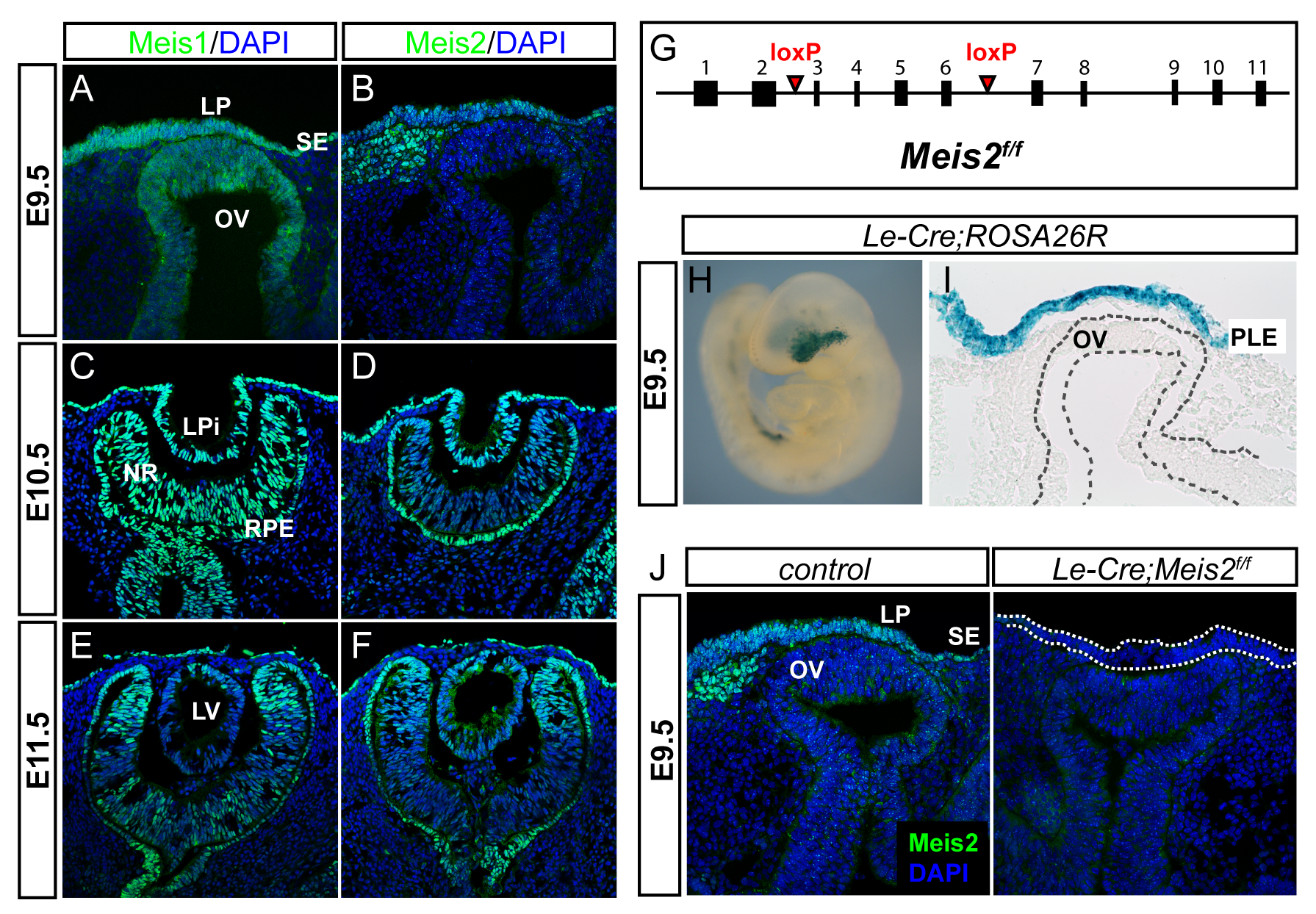

Supplement: S1 Fig — Le-Cre-mediated Meis2 elimination from presumptive lens ectoderm. (A-F) Cryosections from wild-type embryos of the indicated ages labeled for Meis1 and Meis2. (A, B) At E9.5, both Meis1 and Meis2 are expressed in lens placode (LP), and surrounding head surface ectoderm (SE) of wild-type embryo. Meis1 is also detected in optic vesicle (OV) and Meis2 in mesenchymal cells (MC). (C) At E10.5, Meis1 is present in lens pit (Lpi), surrounding SE, neural retina (NR) and retinal pigmented epithelium (RPE). (D) Meis2 expression is present in lens pit, retinal pigmented epithelium and weakly in neural retina. (E) At E11.5 Meis1 expression persists in SE, lens vesicle (LV), RPE and in some cells of NR. (F) Meis2 is detected in SE, LV, RPE and peripheral NR. (G) Schematic representation of targeted Meis2 locus with marked positions of inserted loxP sites. (H, I) Le-Cre activity is demonstrated using the ROSA26R reporter mouse line. Whole-mounts or sections were stained with X-gal at E9.5 to show Cre activity in the eye primordium. (J) Left: Immunofluorescence signal showing Meis2 expression in surface ectoderm (SE) and lens placode (LP) in section of E9.5 control embryo. Right: Region with inactivated Meis2 is indicated with a dashed line in section of E9.5 Meis2 mutant. (TIF) [file pgen.1006441.s001.tif]

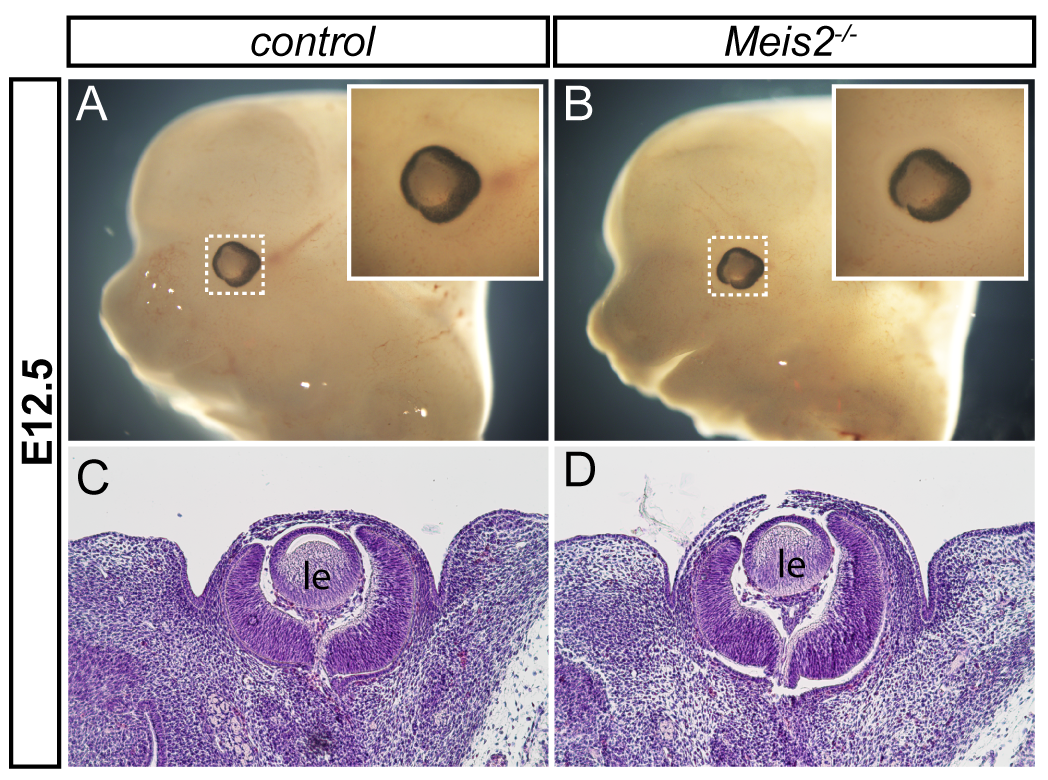

Supplement: S2 Fig — (A, B) External eye of E12.5 Meis2-/- embryo appears comparable to control eye (magnification of eye in insets). (C, D) Hematoxylin-eosin-stained sections at E12.5 do not demonstrate any obvious changes of lens size or morphology in Meis2-/- embryos. le – lens. (TIF) [file pgen.1006441.s002.tif]

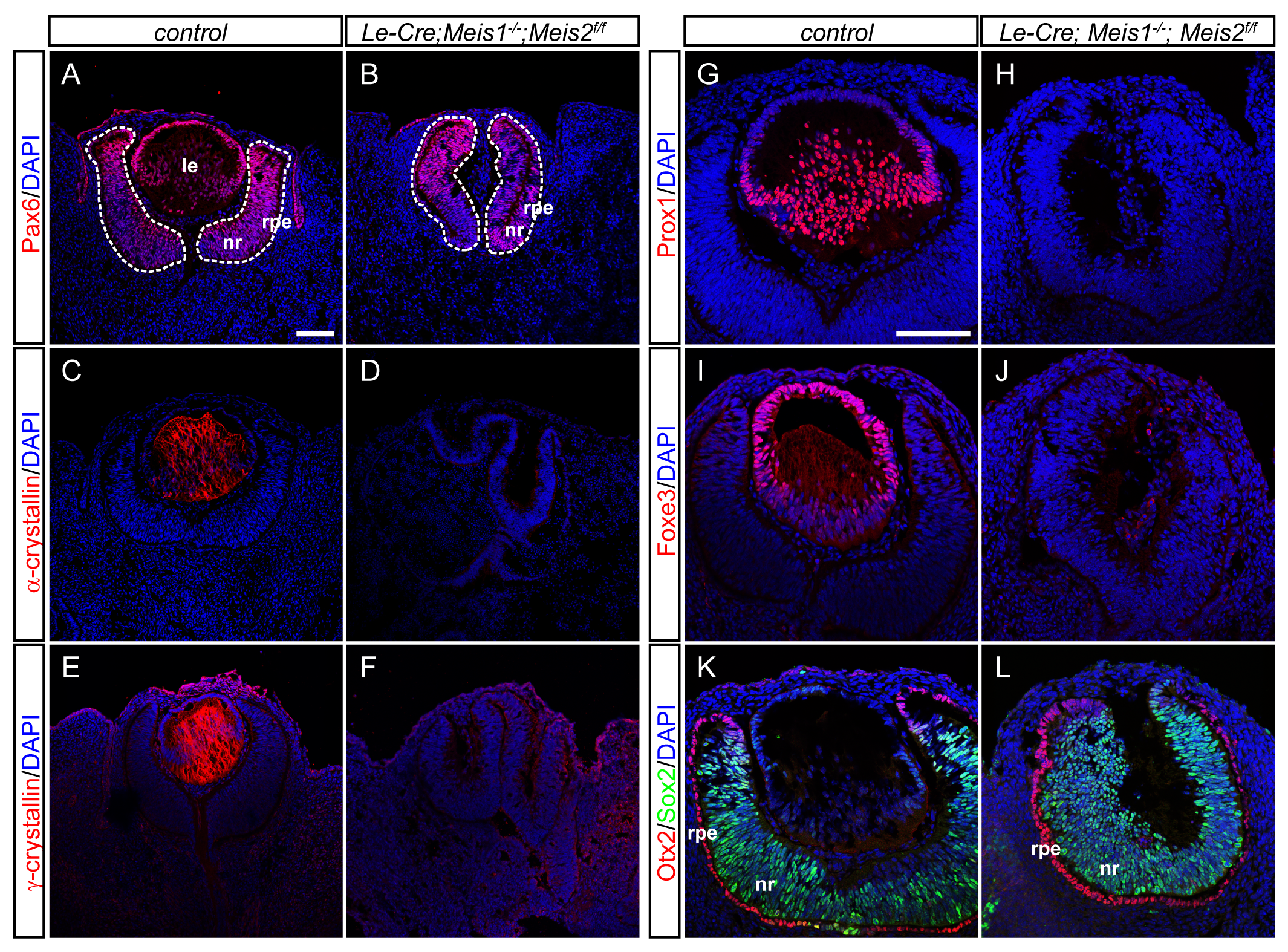

Supplement: S3 Fig — (A-L) Cryosections from E12.5 control and Le-Cre;Meis1-/-;Meis2f/f embryos stained with antibody as indicated, and nuclei counterstained with DAPI. (B) In Meis1/Meis2 double mutants expression of Pax6 is maintained only in neural retina and retinal pigmented epithelium (RPE), since lens is not formed.(D, F, H, J) Note, that lens specific proteins (α- and γ-crystallin, Prox1, Foxe3) are not detected in sections of Le-Cre;Meis1-/-;Meis2f/f embryos. (L) Two separate populations of cells expressing either neural retina (Sox2) or RPE (Otx2) specific markers are detected in Meis1/Meis2 double mutant. Scale bars indicate 100 μm. le-lens, nr-neural retina, rpe-retinal pigmented epithelium. (TIF) [file pgen.1006441.s003.tif]

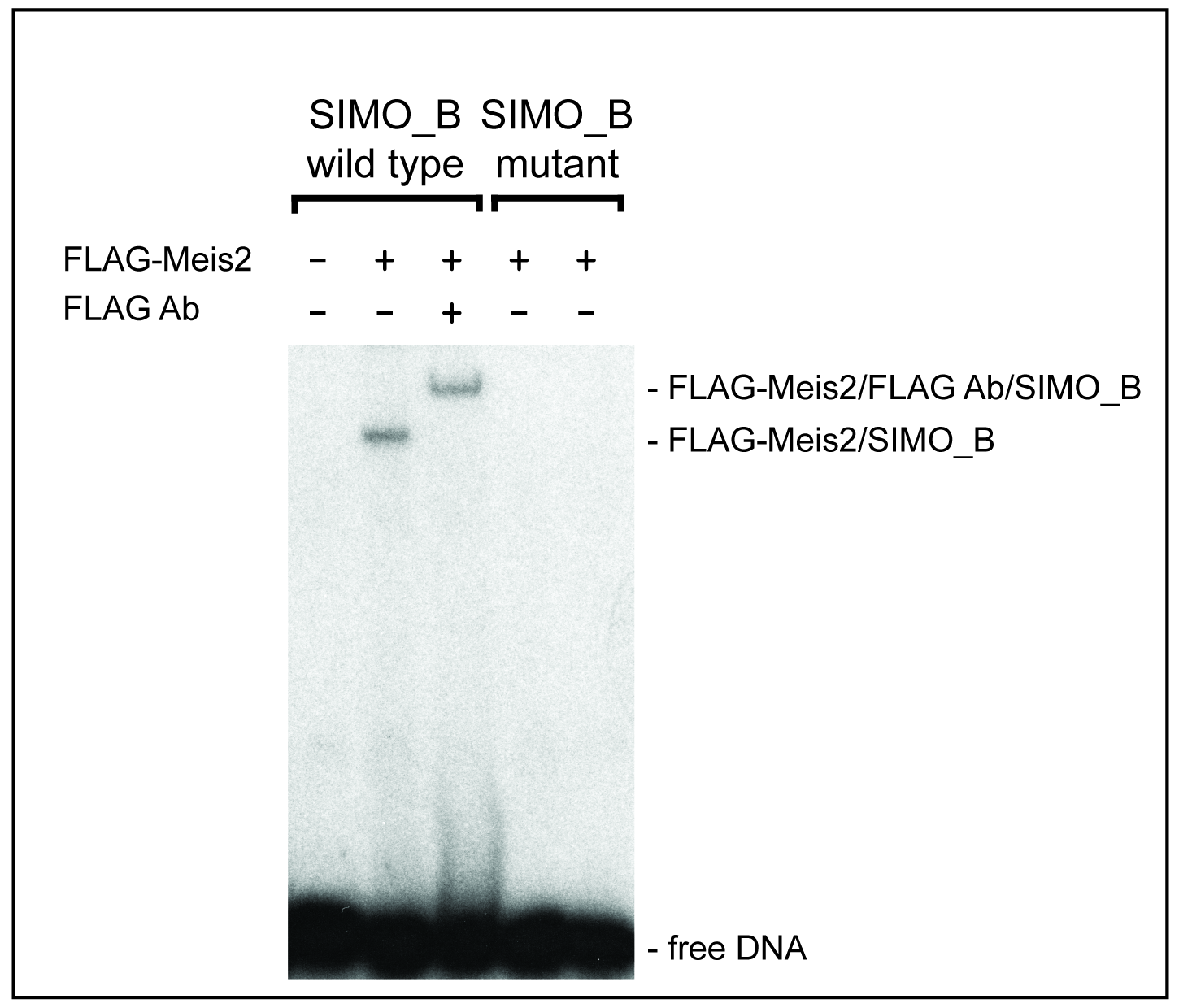

Supplement: S4 Fig — FLAG-tagged Meis2 binds wild-type SIMO_B and can be supershifted by an anti-FLAG antibody. No interaction is detected when a single point mutation is introduced into SIMO_B binding site changing Meis recognition sequence TGACAA into TcACAA. (TIF) [file pgen.1006441.s004.tif]

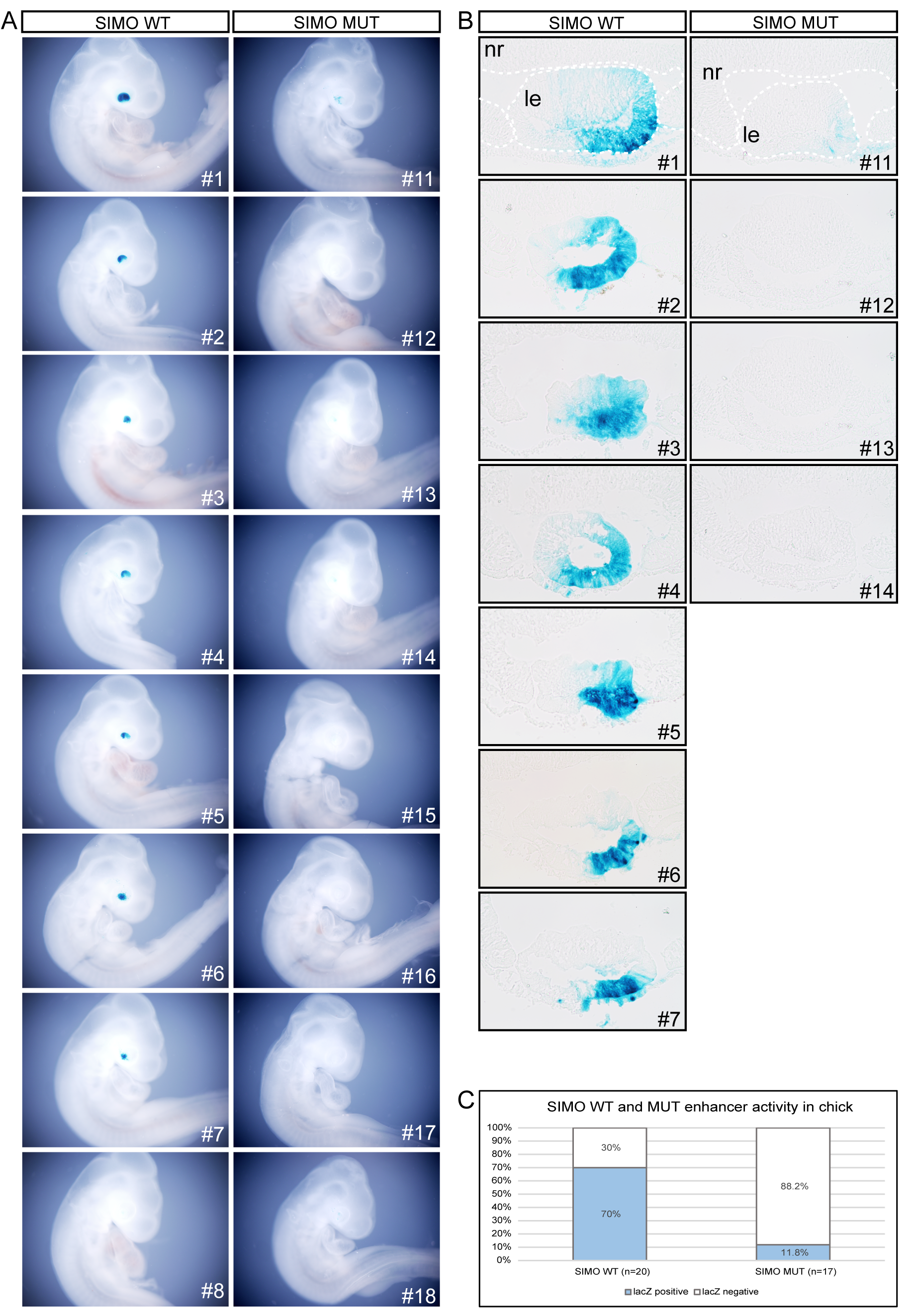

Supplement: S5 Fig — (A) Overview of whole-mount X-gal staining of chick embryos electroporated with reporter construct containing either wild-type or mutant SIMO fragment. (B) Histological sections through the eye of depicted chick embryos. (C) Quantification of positive and negative X-gal (lacZ) staining in electroporated chick embryos. (TIF) [file pgen.1006441.s005.tif]

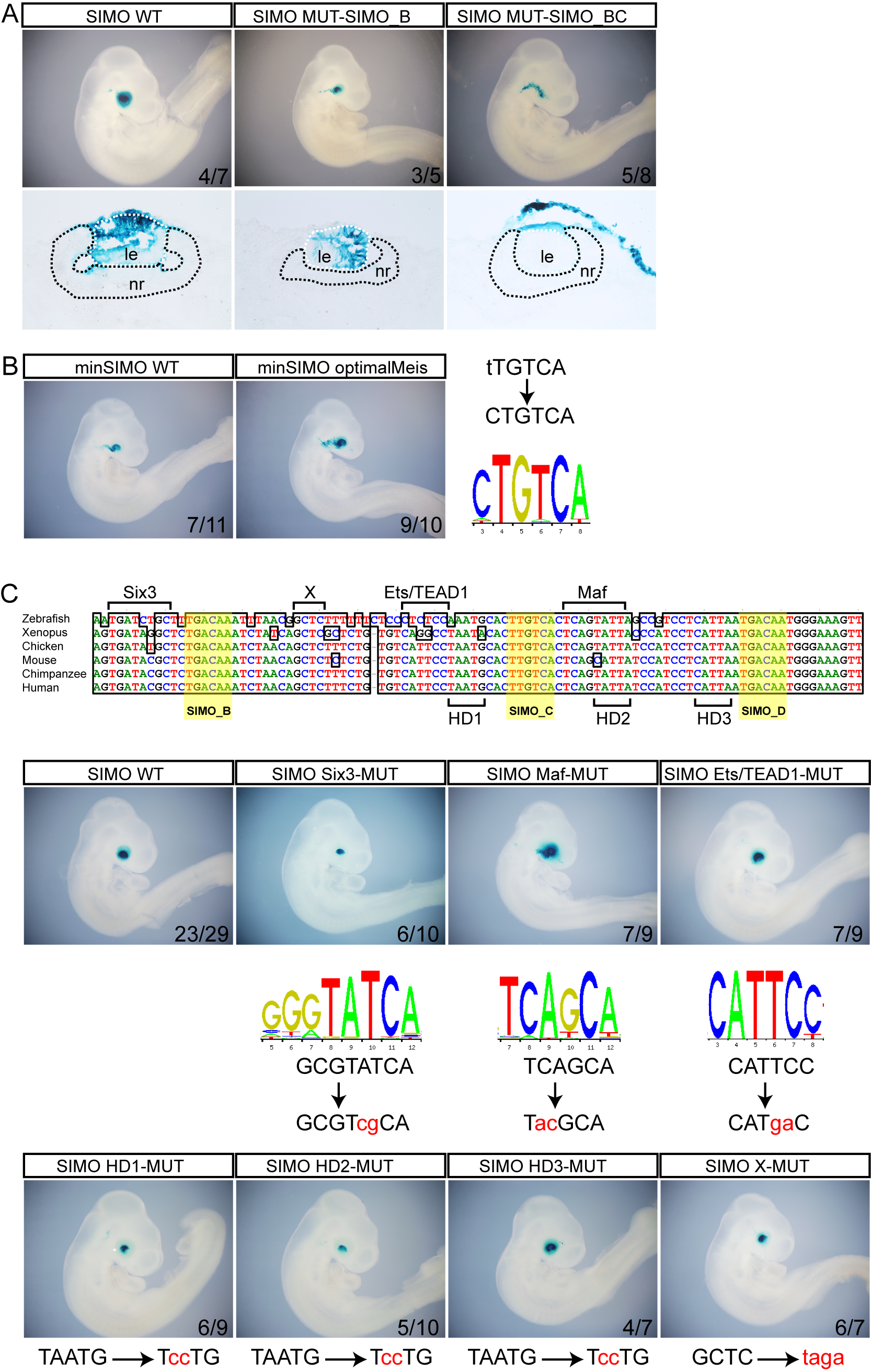

Supplement: S6 Fig — (A-C) Wholemount X-gal stained chick embryos (at HH20-21) showing the expression of lacZ reporter gene under the control of minimal hsp68 promoter fused to wild-type or mutated mouse SIMO electroporated into chick eye forming region at developmental stage HH10-11. The numbers of embryos displaying expression pattern shown are indicated in each panel. (A) Contribution of individual Meis binding sites to SIMO enhancer activity. Reporter gene constructs carrying wild-type SIMO (SIMO WT), SIMO mutated in a single Meis binding site (SIMO MUT-SIMO_B), or two Meis binding sites (SIMO MUT-SIMO_BC) were used for electroporation in ovo. Whole-mount X-gal staining demostrate the effect of mutated Meis binding sites on expression of reporter gene. Cryosections through eye region illustrate a marked decrease of lacZ expression when a single Meis binding site (SIMO_B) was mutated, and a complete loss of lens-specific expression when two Meis binding sites (SIMO_BC) were mutated. (B) Optimized Meis binding sites increase the activity of SIMO enhancer. Reporter gene constructs carrying either minimal wild-type SIMO (minSIMO WT), or minimal SIMO in which natural Meis binding sites TGACAA were substituted with optimized binding sequence TGACAG (minSIMO optimalMeis) were used for electroporation in ovo. Whole-mount X-gal staining shows that the presence of optimized Meis binding sites in SIMO moderately increases the expression of reporter gene. (C) The effect of selected mutations in potential transcription factor binding sites on SIMO enhancer activity. DNA constructs containing either the wild-type SIMO (SIMO WT), or the enhancer carrying mutations in binding sites for the indicated transcription factor were used for electroporation in ovo. Schematic pictures of transcription factor binding motifs are taken from JASPAR database. Mutated nucleotides in binding site of each transcription factor are highlighted in small red letters. nr – neural retina, le – lens. (TIF) [file pgen.1006441.s006.tif]

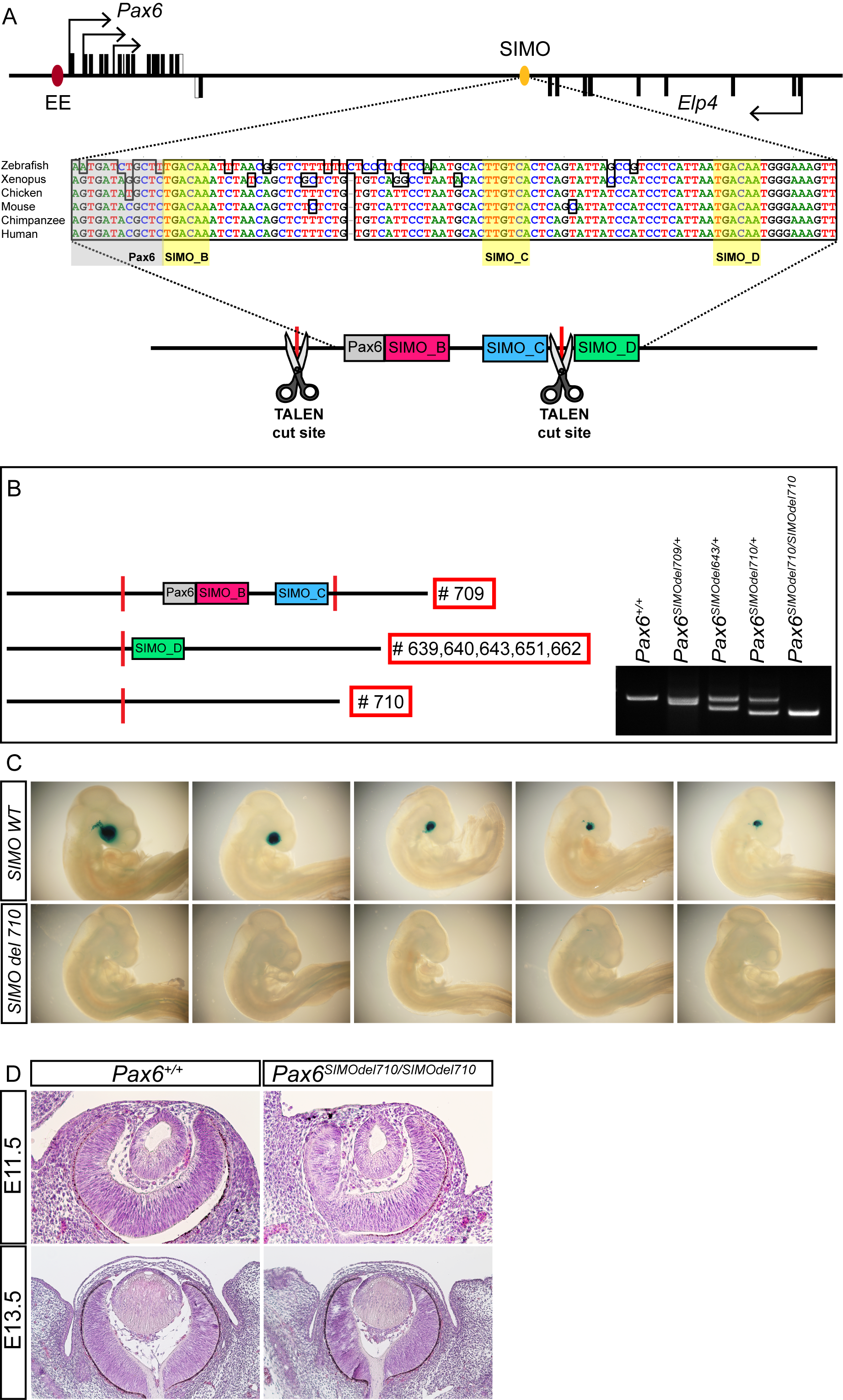

Supplement: S7 Fig — (A) Schematic representation of the Pax6 locus, displaying the exons of Pax6 (black boxes, top strand) and adjacent Elp4 gene (black boxes, bottom strand). Ectodermal enhancer (EE) is indicated with red oval; SIMO enhancer is indicated with yellow oval. The relative position of TALEN recognition sequences is shown with regards to Pax6 autoregulatory element [19], shaded grey and Meis1/2 binding sites SIMO_B, SIMO_C and SIMO_D (all shaded yellow). (B) Schematic representation and PCR genotyping of deletions in individual lines of mice characterized (line number is indicated in red box). (C) Whole-mount view of β-galactosidase–stained chick embryos of stage HH21-22 electroporated either with wild-type or with mutant SIMO carrying a deletion found in line #710. Positive X-gal staining correlates with the activity of reporter constructs. (D) Histological sections of E11.5 and E13.5 control and Pax6SIMOdel710/SIMOdel710 embryonic eyes. (TIF) [file pgen.1006441.s007.tif]

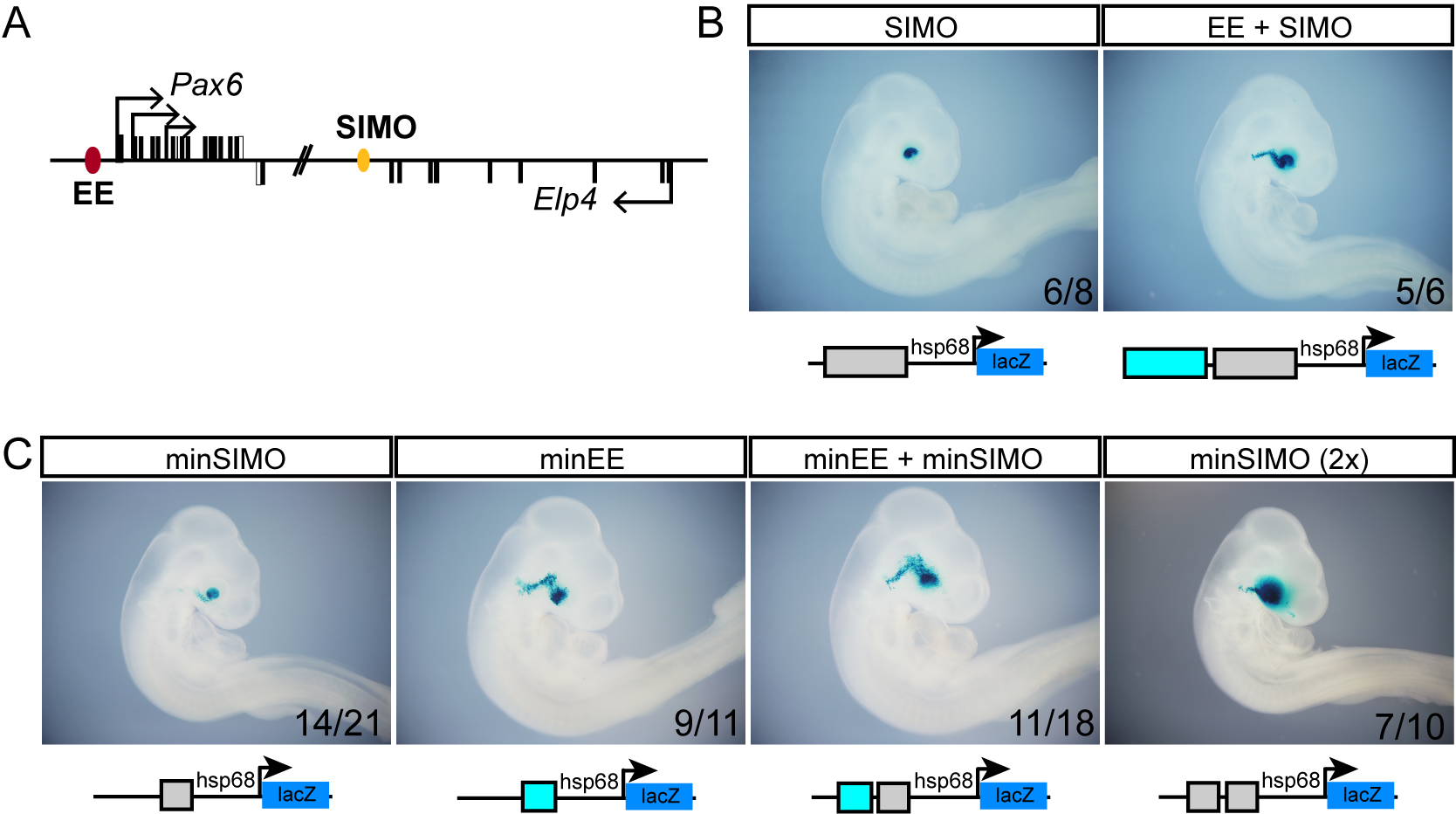

Supplement: S8 Fig — (A) Schematic representation of the Pax6 locus, displaying the exons of Pax6 (black boxes, top strand) and adjacent Elp4 gene (black boxes, bottom strand). Ectodermal enhancer (EE) is indicated with red oval; SIMO enhancer is indicated with yellow oval. (B,C) Reporter gene constructs (depicted with schematic view) carrying either SIMO alone, EE alone, or enhancer combinations were used for electroporation to reveal impact of these Pax6 enhancers for strength and specificity of expression. Combinations of EE and SIMO (EE + SIMO, minEE + minSIMO) ensure stronger expression of reporter gene as compared to SIMO alone or EE alone. While minimal EE (minEE) drives stronger expression of reporter gene than minimal SIMO (minSIMO), the two copies of minSIMO enhancer (minSIMO 2x) provides the strongest reporter gene expression of enhancer variants tested in this experiment. The numbers of embryos displaying expression pattern shown are indicated. (TIF) [file pgen.1006441.s008.tif]

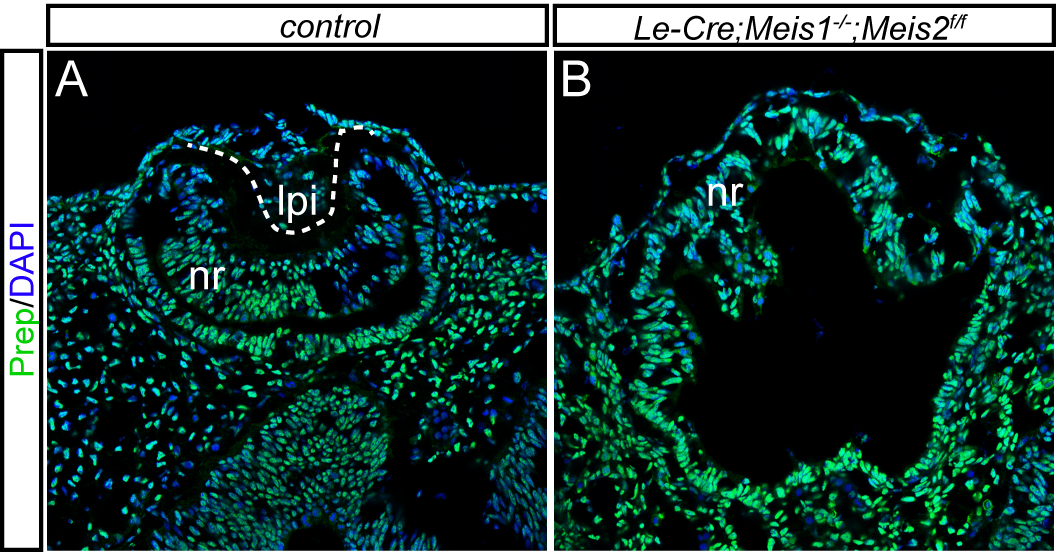

Supplement: S9 Fig — (A, B) Cryosections through eye region of E10.5 control and Le-Cre;Meis1-/-;Meis2f/f embryos stained with anti-Prep antibody, and nuclei counterstained with DAPI. Meis1/Meis2 double mutants did not show changes in Prep expression. nr-neural retina, lpi – lens pit. (TIF) [file pgen.1006441.s009.tif]
