# Supplementary material for: The Gene Regulatory Network of Lens Induction Is Wired through Meis-Dependent Shadow Enhancers of Pax6
Source: PLoS Genet. 2016 Dec 5;12(12):e1006441. doi: 10.1371/journal.pgen.1006441 (PMC5137874; doi:10.1371/journal.pgen.1006441)
Supplement: S1 Table — (DOCX) [file pgen.1006441.s010.docx]

**S1 Table. Oligonucleotides.**

| **Application** | **Sequence (5’-3’)** |
| --- | --- |
| Meis2^f/f^ genotyping | GCAAGGGTGCTGAGGTTAAA |
|  | TCAGACCCAGGAATTTGAGG |
| SIMO deletion genotyping | CATGTACAGTCTGATGACTTTTA |
|  | AGTAGTTCCCGCCTCATGTTT |
| ChIP qRT-PCR SIMO_A | CACTATGTTCACCTCAGGATCAT |
|  | CAAGTGGCAGTTTGGTGC |
| ChIP qRT-PCR SIMO_B/C/D | AATGTCGTCGAGCGTCAG |
|  | GGTCCCATTGTAAAGAGTAGATG |
| ChIP qRT-PCR EE | CTAAAGTAGACACAGCCTT |
|  | GGAGACATTAGCTGAATTC |
| ChIP qRT-PCR Axin2 | GATTGGTGGCTCTTGGTGTT |
|  | CTCTCACTGCTACCCGGT |
| ChIP qRT-PCR Neurod1 | ACAGACGCTCTGCAAAGGTTT |
|  | GGACTGGTAGGAGTAGGGATG |
| Meis SIMO_B wild-type binding site (EMSA) | AGTGATACGCTCTGACAAATCTAACAGCTC |
|  | GAGCTGTTAGATTTGTCAGAGCGTATCACT |
| Meis SIMO_B mutant binding site (EMSA) | AGTGATACGCTCTcACAAATCTAACAGCTC |
|  | GAGCTGTTAGATTTGTgAGAGCGTATCACT |
| Mouse SIMO enhancer (chick electroporation) | CATGTACAGTCTGATGACTTTTAC |
|  | CTTGAGTATTTCAGCACTTTTTCAG |
| Minimal SIMO "minSIMO" (chick electroporation) | GCGTCAGTGCCTGAAGTGATACGCTCTGACAAATCTAACAGCTCTCTCTGTGTCATTCCTAATGCACTTGTCACTCAGCATTATCCATCCTCATTAATGACAATGGGAAAGTTT |
| Minimal EE "minEE" (chick electroporation) | ATGAGAGATCTTTCCGCTCATTGCCCATTCAAATACAATTGTAGATCGAAGCCGGCCTTGTCAGGTTGAGAAAAAGTGAATCTCTAACATCCAGGACGTGCCTGTCTACTTTCA |
| Zebrafish SIMO enhancer (transgenesis) | AGGTGACAGATTTCAGCAGCA |
|  | CTAGTGGCAGATCAAGTGGT |
| EE deletion (sgRNA constructs) | TAGGATGAGGTTGGGGTGCTTC |
|  | AAACGAAGCACCCCAACCTCAT |
|  | TAGGTGGCTTAGTAATTTAAAC |
|  | AAACGTTTAAATTACTAAGCCA |
|  | TAGGAACTAACTGCAAGAATTG |
|  | AAACCAATTCTTGCAGTTAGTT |
|  | TAGGGGAATGTTCTTGAATTGA |
|  | AAACTCAATTCAAGAACATTCC |
| EE deletion genotyping | AAGTCCAGTTGGCAGGTGTC |
|  | GCTGCTCTGCCAGGAAGTAA |
