# Supplementary material for: The Gene Regulatory Network of Lens Induction Is Wired through Meis-Dependent Shadow Enhancers of Pax6
Source: PLoS Genet. 2016 Dec 5;12(12):e1006441. doi: 10.1371/journal.pgen.1006441 (PMC5137874; doi:10.1371/journal.pgen.1006441)
Supplement: S2 Table — (DOCX) [file pgen.1006441.s011.docx]

**S2 Table. Primary antibodies.**

| **Antibody** | **Host** | **Dilution** | **Source** |
| --- | --- | --- | --- |
| **Pax6** | rabbit | 1:1000 | Covance (PRP-278P) |
| **Pax6** | mouse | 1:3000 | DSHB |
| **Prox1** | rabbit | 1:2000 | Merc Millipore AB5474 |
| **Prox1** | rabbit | 1:1000 | Abcam 11941 |
| **Meis1** | rabbit | 1:1000 | Kind gift from A. Buchberg |
| **Meis2** | rabbit | 1:1000 | Kind gift from A. Buchberg |
| **Foxe3** | rabbit | 1:500 | Kind gift from P. Carlsson |
| **Six3** | rabbit | 1:3000 | Kind gift from P. Bovolenta (unpublished) |
| **Sox2** | goat | 1:400 | Santa Cruz (sc-17320) |
| **α-crystallin** | rabbit | 1:500 | Kind gift from S. Ziegler |
| **γ-crystallin** | rabbit | 1:500 | Kind gift from H. Kondoh |
| **Otx2** | rabbit | 1:300 | R&D Systems (BAF1979) |
| **Prep** | mouse | 1:300 | Santa Cruz (sc-81977) |
